# The experiences of reproductive concerns in cancer survivors: A systematic review and meta‐synthesis of qualitative studies

**DOI:** 10.1002/cam4.6531

**Published:** 2023-12-09

**Authors:** Ying Dong, Zhenyu Yue, Huan Zhuang, Chen Zhang, Yu Fang, Guichun Jiang

**Affiliations:** ^1^ LiaoNing Cancer Hospital & Institute, DaLian Medical University School of Nursing Shenyang China; ^2^ LiaoNing Cancer Hospital & Institute Shenyang China; ^3^ Third Department of Gynecology LiaoNing Cancer Hospital & Institute Shenyang China; ^4^ DaLian Medical University School of Nursing Dalian China; ^5^ Clinical Skills Training Center LiaoNing Cancer Hospital & Institute Shenyang China

**Keywords:** cancer, meta‐synthesis, reproductive concerns, systematic review

## Abstract

**Aim:**

The aim of this study was to synthesize qualitative research evidence on cancer survivors' experiences with reproductive concerns (RC).

**Methods:**

We conducted a systematic search of qualitative studies and utilized the meta‐aggregation approach. The database searches were extended up to May 14, 2023, encompassing 12 databases, specifically MEDLINE, CINAHL, PubMed, EMBASE, Scopus, Web of Science (Core Collection), AMED, PsycINFO, The Cochrane Library, CNKI, Wan Fang Data, and VIP.

**Results:**

Three overarching themes were synthesized from the analysis of 21 studies that explored cancer patients' awareness of reproductive concerns, their perceptions, needs, and coping styles. These themes encapsulate the multifaceted aspects of cancer patients' reproductive concerns: “Gender differences in fertility concerns among cancer patients: Perspectives from men and women”; “The influence of age: Experiences with fertility issues among cancer patients at different life stages”; “The impact of treatment stages on fertility concerns: The evolution of perception and coping strategies in the course of cancer treatment”.

**Conclusion:**

Our study presents an in‐depth exploration of the reproductive concerns experienced by cancer patients from various perspectives. We found that the internal experiences of reproductive concerns, their perceptions, needs, and coping mechanisms differ based on their roles. This comprehensive understanding of the complex emotions and needs of cancer patients when confronted with fertility issues can guide clinicians in providing more effective medical assistance, psychological counseling, and fertility‐related information services.

## 1 INTRODUCTION

The International Agency for Research on Cancer (IARC) released “GLOBOCAN 2020: Global Cancer Incidence and Mortality Forecast,” indicating an estimated 19.29 million new cancer cases and 10 million cancer deaths worldwide in 2020.^1^, ^2^ By 2040, the global cancer burden is projected to increase by 50% compared to 2020, with nearly 30 million new cancer cases expected.^3^ Advances in medical technology, improved early detection and screening, and emerging novel treatments such as targeted therapy and immunotherapy have enhanced personalized and precision medicine. As a result, despite an increase in cancer patient numbers, survival rates have markedly improved.^4^, ^5^, ^6^ Over the past decade, the survival rate of cancer patients in China has gradually increased. The current 5‐year relative survival rate of cancer patients in China is approximately 40.50%, an overall increase of approximately 10% points compared to a decade ago.^7^

As cancer patients continue to be diagnosed at younger ages and survival periods lengthen, many cancer patients who have not yet completed their family planning may immediately face considerations about their reproductive potential upon receiving their diagnosis.^8^, ^9^ Treatment methods such as surgery, chemotherapy, or radiation therapy can directly or indirectly impact their reproductive organs or functions, thereby increasing the challenges of fertility or leading to infertility.^10^, ^11^ For young cancer patients, building a family often becomes their primary focus. The growing desire for parenthood among cancer patients further underscores the significance of reproductive issues. The concept of reproductive concerns encompasses the excessive worries about fertility and parenting stemming from uncertainties about fertility and a lack of relevant reproductive information due to cancer and its treatments.^12^ More specifically, reproductive concerns refer to the excessive apprehension experienced by individuals who have not fulfilled their family planning due to the uncertainties and insufficient information about fertility resulting from cancer and its treatments. This includes concerns about fertility capacity, personal health, child health and care, partner relationships, acceptance of pregnancy and infertility, and more.^12^ Studies indicate that over 75% of young cancer patients who have not yet experienced childbirth express a desire to have children after treatment, and patients who have already given birth wish to do so again.^13^ A study examining reproductive issues in young cancer survivors^14^ found that the occurrence rate of reproductive concerns ranged from 44% to 86%, with 28% to 44% of patients experiencing moderate to severe reproductive problems. Research indicates that the presence of fertility issues increases the likelihood of patients developing depression,^15^, ^16^ profoundly affecting their physical and emotional well‐being, along with their overall quality of life.^15^, ^17^ The severity of reproductive concerns is positively associated with depression risk.^15^, ^16^ Moreover, reproductive concerns can result in negative perceptions of body image,^18^ decreased self‐disclosure,^19^ impaired sociopsychological adaptation,^20^ impact treatment choices,^21^, ^22^, ^23^ trigger post‐decision regret, lower patients' levels of hope and treatment compliance,^24^ ultimately diminishing patients' overall quality of life.^15^

Currently, qualitative research on fertility concerns primarily focuses on exploring the inner experiences of reproductive‐aged female cancer patients,^25^ especially in China.^26^, ^27^, ^28^ Undoubtedly, fertility exerts a significant impact on young women, however, the reproductive needs of other cancer patients who are not often included in discussions cannot be overlooked, such as reproductive‐aged male patients and pediatric and adolescent cancer patients. Data show that globally, approximately two‐thirds of childhood and adolescent cancer survivors experience long‐term side effects following treatment, and one common delayed effect is infertility.^29^, ^30^, ^31^ More than 50% of young male cancer patients experience impaired fertility after treatment, with some even facing a lifelong risk of infertility. The severity of this harm depends largely on factors like cancer type, treatment protocols, radiation and chemotherapy dosages, and the utilization of fertility preservation measures.^32^ This reveals that both male cancer patients and pediatric and adolescent cancer patients are equally afflicted by fertility concerns. It is necessary to compare and integrate the inner experiences of all patients with reproductive needs to better guide the development of clinical practices.^33^, ^34^, ^35^, ^36^, ^37^ However, a considerable portion of qualitative research has not comprehensively addressed the limitations brought about by exploring a single gender or age group. Moreover, patients in different stages of treatment still possess distinct treatment needs. Logan et al^38^ through a systematic review of fertility‐related interventions adopted by cancer patients at different phases of tumor treatment (diagnosis, treatment, and survivorship), found that during diagnosis and treatment, primary interventions involve psychological counseling or fertility consultations and support. Fertility concerns persist during survivorship, and interventions like fertility treatment and care for tumor fertility can be implemented at this stage. Fertility concerns exist from the onset of cancer diagnosis and persist through a significant portion of survivorship, and the levels and causes of fertility concerns vary at different time periods,^38^ resulting in differing corresponding interventions. Therefore, an in‐depth study of the inner experiences of cancer patients' fertility concerns during different treatment stages will provide better clinical guidance and a deeper understanding of patients' fertility issues, thereby facilitating the implementation of subsequent intervention measures.

In view of these considerations, this study employs a comprehensive research approach that systematically collates all qualitative research relevant to cancer patients' fertility concerns. It encompasses the inclusion of gender, age, and various stages of cancer treatment, thereby constructing a more comprehensive and in‐depth research framework. Additionally, this study critically analyzes patients' cognitive attitudes, needs, and coping strategies from different perspectives, offering a novel viewpoint for a profound understanding of cancer patients' inner experiences when facing fertility concerns. To the best of our knowledge, such research has not been previously identified. This research provides theoretical support for gender, age, and treatment stage considerations in clinical psychological support. It aids in better comprehending and supporting the unique fertility needs of this specific group, thereby offering more targeted psychological support and counseling services. By comparing patients of different genders, ages, and treatment stages, we can design more personalized intervention strategies to meet the diverse needs of individual patients. This approach increases societal awareness of fertility concerns among cancer patients, facilitates effective doctor–patient communication, encourages policymakers and non‐profit organizations to provide more policy and resource support, and enhances patients' overall quality of life. In summary, the primary focus of this study is to delve into the inner experiences of cancer patients' fertility concerns. Specifically, we investigate how cognitive attitudes, needs, and coping strategies related to fertility concerns vary across different genders, ages, and stages of cancer treatment. This study aims to shed light on the specific differences and disparities among these groups.

## 2 METHODS

### 2.1 Aim

To systematically review and synthesize qualitative research exploring the evidence on the cancer survivor's experience with reproductive concerns. The review question was as follows:

Understand the experience of cancer patients of different genders and roles when facing fertility problems; ways to cope with reproductive concerns of patients in different periods of anticancer treatment.

### 2.2 Design

The systematic review was conducted in accordance with the guidelines of the Joanna Briggs Institute Reviewer's Manual.^39^ Enhancing transparency in reporting, the synthesis of qualitative research (ENTREQ) statement was used to facilitate reporting.^40^

### 2.3 Search methods

#### 2.3.1 Registration PROSPERO CRD42023426230

#### 2.3.2 Search strategy

In pursuit of primary research studies, we initially executed a cursory search in both MEDLINE and CINAHL, the purpose of which was to identify subject words and keywords for inclusion in our search strategy. Subsequently, the text words extracted from the titles, abstracts, and index terms served as the foundation for the development of a comprehensive search strategy across all databases, including MEDLINE, CINAHL, PubMed, EMBASE, Scopus, Web of Science, AMED, and psycINFO, The Cochrane Library, CNKI, Wan Fang Data, and VIP. This strategy was then duly refined and implemented. As a final measure, we meticulously reviewed the reference lists of articles chosen for data extraction in an endeavor to uncover additional studies. The literature search for data inclusion in this study encompassed the period up until May 14, 2023, ensuring the inclusion of current and up‐to‐date literature from databases.

### 2.4 Inclusion/exclusion criteria

The inclusion criteria were as follows: (1) Participants were patients diagnosed with cancer who expressed concerns about fertility. The patient population spanned all ages, cultures, ethnicities, genders, and disease stages. Studies focusing on patients without fertility concerns or individuals who had fertility concerns but were not diagnosed with cancer were excluded. (2) Studies were included if they reported on the perceived needs and coping mechanisms of patients of different gender roles at various stages of cancer treatment when faced with fertility issues. Studies were excluded if they merely discussed the concept or theory of fertility issues in cancer patients without delving into their perceived needs and coping mechanisms. (3) We considered all environments and geographical areas that might involve cancer patients with fertility concerns. These environments included but were not limited to home care, hospitals, and specialized clinics. (4) Types of publications included peer‐reviewed journal articles. The types of studies included primary qualitative research studies that reported on the inner experiences of cancer patients facing fertility issues, presenting direct quotes from these patients. We included studies of all age ranges and qualitative methodologies. The studies must have been presented in English or Chinese or have a translation available. Publications in languages other than English or Chinese, duplicate publications, conference articles, and those where full text was not available were excluded.

### 2.5 Study screening and selection

Two researchers independently searched each database according to the established inclusion/exclusion criteria and search strings. The search results were then imported into Endnote X 9.3.3 for the removal of duplicates. Following this, a second round of selection was conducted based on the titles and abstracts of the articles. The remaining articles were further screened by full‐text reading. Lastly, the references of the included articles were thoroughly reviewed to include any additional articles that meet the study inclusion/exclusion criteria.

### 2.6 Quality appraisal

The quality of the selected studies was evaluated based on a checklist for qualitative research provided by the Joanna Briggs Institute Reviewer's Manual.^41^ This appraisal was performed independently by two reviewers, and any differences in opinions were reconciled by a third reviewer.

### 2.7 Data abstraction

Two independent reviewers meticulously gathered and systematized an array of information from the chosen scholarly articles, encompassing authorship, country of origin, publication date, research design methodology, methods for data acquisition, specified treatment phases (grounded in the established inclusion criteria), phenomena under investigation, participant demographics (including mean age, sample size, and location of recruitment), along with key findings of the study. Should inconsistencies or conflicts arise in this progression, they were addressed and reconciled through collaborative dialogue and discussion among the reviewers.

### 2.8 Data synthesis

In the process of synthesizing the research findings, we employed a method of meta‐aggregation and utilized the Joanna Briggs Institute System for the Unified Management, Assessment, and Review of Information (JBI‐SUMARI) for data synthesis.^41^ This process was delineated into four distinct steps, with each step being carefully considered by our team to ensure the accuracy and credibility of the synthesized results. (a) Initially, pertinent quotations and statements closely aligned with our research objective were selected by one reviewer and further scrutinized by another to ensure their alignment with the research aim. (b) To uphold methodological rigor, particular emphasis was placed on the assignment of credibility levels (unequivocal, credible, or unsupported), a step carried out independently by two reviewers. In cases where multiple quotations described the same finding, credibility levels were assigned based on the established hierarchy (unequivocal > credible > unsupported) to maintain consistency between the quotations and the findings. (c) Subsequently, findings rated as unequivocal or credible were categorized and integrated based on their content similarity. Conversely, findings deemed unsupported were excluded from further analysis. (d) We then amalgamated these categorized findings into comprehensively synthesized results. Prior to this amalgamation, the logical coherence and reliability of each step were ensured through collaborative team efforts and discussions. Ultimately, an evaluation of the relevance and applicability of the synthesized results was conducted by all authors to ensure the rigor and effectiveness of the entire process.

### 2.9 Confidence in the findings

We used the JBI approach (ConQual) to evaluate the confidence in the outcomes of our synthesized research.^41^ ConQual is a useful tool for gauging the trustworthiness of qualitative study results and can aid in the process of clinical decision‐making. The assurance in the findings (rated as high, moderate, low, or very low) was decided based on the dependability and credibility of the included studies. For each study, if four or five criteria were met, the synthesized outcomes remained at their current level. If only two or three criteria were satisfied, the confidence level decreased by one. If less than two criteria were met, the ratings dropped by two levels. The dependability of synthesized outcomes was based on the collective level of dependability across the included findings. The credibility levels of the synthesized outcomes were drawn from the credibility levels of the original findings. If the synthesized outcomes contained a blend of unequivocal (U) and credible findings (C), the credibility of the synthesized findings was downgraded by one level.

## 3 RESULTS

### 3.1 Search outcome

**FIGURE 1:**
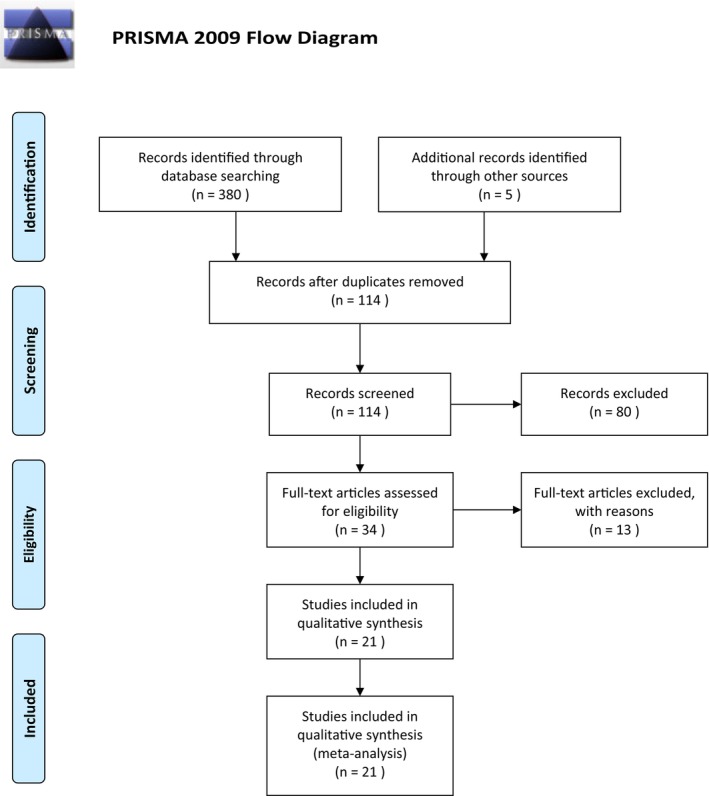
PRISMA 2009 flow diagram. From: Moher D, Liberati A, Tetzlaff J, Altman DG, The PRISMA Group (2009). Preferred Reporting Items for Systematic Reviews and Meta‐Analyses: The PRISMA Statement. PLoS Med 6(7): e1000097. 10.1371/journal.pmed1000097. For more information, visit www.prisma‐statement.org.

The study search took place in May 2023. Database searches yielded 380 articles, and 5 additional articles were sourced from included studies reference lists. Duplicates were removed, and 114 articles remained. Thirty‐four full texts were screened and of those, 21 met the selection criteria.^33^, ^34^, ^35^, ^36^, ^42^, ^43^, ^44^, ^45^, ^46^, ^47^, ^48^, ^49^, ^50^, ^51^, ^52^, ^53^, ^54^, ^55^, ^56^, ^57^, ^58^ Figure 1 shows a flow chart of the search screening process. The majority of studies that were excluded during the title and abstract screening were due to the population or study design being outside of the selection criteria. The articles excluded during the full‐text review were primarily due to their research results not being applicable, the low quality of the articles, and conference abstracts.

### 3.2 Study characteristics

The methodological approaches of the eight included studies were heterogeneous and included a descriptive phenomenological approach (*n* = 4),^42^, ^43^, ^44^, ^45^ grounded theory (*n* = 3),^33^, ^49^, ^57^ qualitative interpretive description (*n* = 1),^58^ constructivist qualitative study (*n* = 1),^52^ exploratory qualitative study(*n* = 1),^56^ and qualitative descriptive study (*n* = 11).^34^, ^35^, ^36^, ^46^, ^47^, ^48^, ^50^, ^51^, ^53^, ^54^, ^55^ Most studies drew on thematic analysis to construct themes. Studies were set in China (*n* = 4),^42^, ^43^, ^44^, ^45^ Iran (*n* = 1),^46^ United States (*n* = 3),^33^, ^47^, ^48^ Canada (*n* = 2),^34^, ^49^ Brazil (*n* = 1),^35^ Australia (*n* = 6)^36^, ^50^, ^51^, ^52^, ^53^, ^54^ and United Kingdom (*n* = 4).^55^, ^56^, ^57^, ^58^ The age range of the included study population ranged from 0 to 58 years, encompassing a total of 992 patients. Data collection methods included semi‐structured interviews, depth interviews, open‐ended questions, focus group discussions, cognitive debriefing interviews, and narrative interviews. In the included population of cancer patients, breast cancer patients were the majority. Regarding the gender of the cancer patients, female reports were more common. Study characteristics are reported in Table A1.

### 3.3 Quality appraisal

Table 1 displays the outcomes of the quality assessment applied to the included studies. An average rating of “unclear” was assigned in three evaluation criteria among the 21 studies. Statements on authors' philosophical perspectives were avoided in all included studies. Merely Three studies^42^, ^46^, ^50^ specified the researchers' identities in a cultural or theoretical sense. A single study^49^ mentioned the potential influence of the researcher's role on interpreting the findings.

**TABLE 1:** Results of the quality appraisal.

No	Study (year), country	C1	C2	C3	C4	C5	C6	C7	C8	C9	C10
1	Xiao et al (2023), China^42^	U	Y	Y	Y	Y	Y	U	Y	Y	Y
2	Bin (2022), China^43^	U	Y	Y	Y	Y	U	U	Y	Y	Y
3	Xiao (2021), China^44^	U	Y	Y	Y	Y	U	U	Y	Y	Y
4	Tang (2020), China^45^	U	Y	Y	Y	Y	U	U	Y	Y	Y
5	Azizi et al (2023), Iran^46^	U	Y	Y	Y	Y	Y	U	Y	Y	Y
6	Carr (2022), USA^47^	U	Y	Y	Y	Y	U	U	Y	Y	Y
7	Benedict (2016), USA^33^	U	Y	Y	Y	Y	U	U	Y	Y	Y
8	Quinn (2013), USA^48^	U	Y	Y	Y	Y	U	U	Y	Y	Y
9	Vanstone (2021), Canada^49^	U	Y	Y	Y	Y	U	Y	Y	Y	Y
10	Newton (2021), Canada^34^	U	Y	Y	Y	Y	U	U	Y	Y	Y
11	Jardim (2021), Brazil^35^	U	Y	Y	Y	Y	U	U	Y	Y	Y
12	Wang (2020), Australia^50^	U	Y	Y	Y	Y	Y	U	Y	Y	Y
13	Ussher (2018), Australia^36^	U	Y	Y	Y	Y	U	U	Y	Y	Y
14	Perz (2014), Australia^51^	U	Y	Y	Y	Y	U	U	Y	Y	Y
15	Dryden (2014), Australia^52^	U	Y	Y	Y	Y	U	U	Y	Y	Y
16	Kirkman (2013), Australia^53^	U	Y	Y	Y	Y	U	U	Y	Y	Y
17	Penrose (2012), Australia^54^	U	Y	Y	Y	Y	U	U	Y	Y	Y
18	Corney (2014), UK^55^	U	Y	Y	Y	Y	Y	U	Y	Y	Y
19	Crawshaw (2010), UK^56^	U	Y	Y	Y	Y	U	U	Y	Y	Y
20	Crawshaw (2009), UK^57^	U	Y	Y	Y	Y	U	U	Y	Y	Y
21	Chapple (2007), UK^58^	U	Y	Y	Y	Y	U	U	Y	Y	Y

Abbreviations: C1, congruity between the stated philosophical perspective and the research methodology; C2, congruity between the research methodology and the research question or objectives; C3, congruity between the research methodology and the methods used to collect data; C4, congruity between the research methodology and the representation and analysis of data; C5, congruity between the research methodology and the interpretation of the results; C6, identifying the researcher culturally or theoretically; C7, influence of the researcher on the research; C8, representation of participants and their voices; C9, ethical approval by an appropriate body; C10, relationship of the conclusions with the analysis or interpretation of the data; Y, yes; N, no; U, unclear; NA, not applicable.

### 3.4 Review findings

Table A2 provides an overview of all the findings and synthesized findings. Three synthesized findings were identified from 157 findings and 8 categories across the 21 studies. Of these, 145 were rated as unequivocal, and 12 were rated as credible.

#### 3.4.1 Synthesized finding

Theme 1.*“Gender differences in fertility concerns among cancer patients: Perspectives from men and women.”*

Sub‐theme 1.1. “Dual perspectives: Unifying the distinctive experiences of men and women in facing fertility issues”

Sub‐sub‐theme 1.1.1 Men

We extracted the theme of men's fertility concerns from three academic articles, which revealed that men's worries about fertility issues span across physiological, psychological, and social dimensions. Specifically, this encompasses compromised fertility function on a physical level,^33^, ^36^, ^58^ emotional distress where individuals may have confidence in preserving future fertility while simultaneously experiencing psychological anguish,^47^, ^49^, ^52^, ^56^ and potential societal and cultural challenges that could lead to a loss of male identity.^53^, ^56^ Another two articles^56^, ^58^ provided the theme of men's needs when facing fertility issues, with these men desiring professional advice. Lastly, two articles^34^, ^57^ elaborated on the ways men cope with fertility concerns, including the utilization of strategies such as sperm storage.

Collectively, these studies suggest that fertility issues impose profound impacts on men, affecting their identity, emotional experiences, and societal roles. Increased support and resources are needed in their journey of addressing fertility problems.

Sub‐sub‐theme 1.1.2 Women

Several studies^42^, ^44^, ^45^, ^46^, ^48^ have analyzed the negative emotional experiences of female cancer patients facing fertility issues, such as anxiety, self‐doubt, regret, self‐blame, stress, guilt, challenges in dating and partner relationships, health risks, and personal narratives associated with fertility. Other research^33^, ^34^, ^36^ has illuminated the impact of infertility on the life course and identity. Further works^36^, ^46^, ^51^, ^52^ emphasize the multiple manifestations caused by fertility issues, such as changes in body image and feminine identity like hair loss, weight fluctuation, and premature aging, underscoring the importance of fertility.^53^, ^54^ When making decisions about fertility, patients may struggle to make informed choices due to a lack of relevant information.^42^, ^45^, ^51^, ^54^ Regarding the personal needs of female cancer patients, some research^44^, ^46^, ^50^, ^54^ highlights concerns about disease prognosis and survival, fertility and breastfeeding, sex life, fertility preservation decisions, spirituality, treatment, and support. The need for information support about fertility technology protection and the timing and safety of pregnancy and childbirth has also been revealed.^42^, ^44^, ^54^ Additionally, there is a desire for communication, support, and acquisition of diverse scientific reproductive knowledge.^34^, ^45^, ^53^ These encompass a range of information needs, including but not limited to the requirement for expertise from health care professionals^36^, ^44^, ^45^, ^46^; the necessity for scientifically valid fertility information^36^, ^42^, ^44^; the demand for diverse sources of information support^42^, ^44^, ^45^; the need for information regarding potential fertility loss^45^, ^49^; the absence of decision‐making and fertility‐related information^36^, ^52^, ^57^, ^58^; and matters concerning pregnancy post‐chemotherapy and radiotherapy.^58^

Strategies for dealing with fertility issues include accepting reality,^48^, ^51^ seeking support—medical, informational, family, and social,^42^, ^43^, ^50^ changing bad lifestyle habits,^43^, ^48^ handling distress,^48^, ^51^, ^56^ finding meaning,^47^, ^48^ maintaining hope while suffering,^33^, ^56^ and providing advice to health care providers.^50^, ^53^

Collectively, these studies illuminate the complex experiences of women, particularly those with cancer, when grappling with fertility issues. They reveal a range of negative emotions, underscore the need for information and support, and point to effective coping strategies. These insights call for comprehensive and gender‐sensitive approaches to addressing fertility concerns. Specifically, health care providers should be well‐equipped to offer professional advice tailored to women's distinctive needs. Additionally, patients should be empowered with the necessary information and resources to make informed decisions about their fertility. Efforts should also be made to destigmatize discussions around fertility issues, promote open conversations, and reduce psychological distress. Furthermore, a broader societal effort is needed to provide robust support mechanisms and resources for individuals facing these challenges.

Sub‐theme 1.2. “Men vs Women: Dissecting the gender disparity in the perception and management of fertility problems”

Sub‐sub‐theme 1.2.1 “Biology dictates: Unraveling gender differences in fertility.”

From eight original qualitative studies, we identified and integrated different thinking paths in fertility preservation decisions due to physiological differences between men and women.^33^, ^34^, ^35^, ^36^, ^50^, ^56^, ^57^, ^58^ Among them, five studies pointed out that the fertility preservation process for men is relatively straightforward, storing sperm in a sperm bank, making it easier for them to understand and accept this process.^33^, ^35^, ^36^, ^57^, ^58^ In contrast, according to the descriptions in four literature pieces, the fertility preservation process for women is much more complex, involving egg retrieval and freezing. Influenced by physiological cycles, they may require hormone stimulation and surgical intervention. In addition, while preservation has a relatively small impact on the body, women need to conceive within a certain period after the technology implementation; therefore, women may display more emotional responses and hesitations during this decision‐making process.^34^, ^36^, ^50^, ^56^ Another four pieces propose that women are more likely to be affected by emotional fluctuations in their psychological state, whereas men might be more inclined toward rational analysis.^33^, ^34^, ^35^, ^57^ These subjective and objective factors together result in gender differences in fertility preservation decisions. A deep understanding of these physiological and psychological factors will help us provide more targeted fertility preservation advice and services for men and women.

“All found the timing difficult, although understandable. Those offered egg freezing were all facing challenging surgery, including possible limb amputation and loss of reproductive organs. All refused, primarily to avoid delays in treatment. All were pleased to have had the offer but reflected that the resulting impaired fertility of two and the unknown status of the other led to times of later preoccupation with the circumstances of their decision.” (P.385)^57^

“Yeah, I didn't really think about it, to be honest. I just said yeah pretty much on the spot, so it just seemed like commonsense to be honest… cos if I didn't [bank] and it came out at the end of treatment that I was infertile then, I dunno, it would be a bugger, really, yeah, er, cos like in later life if you ever wanted to have children, like you wouldn't have anything there, so.” (P.385)^57^

This content integrates the influence of biological gender differences in fertility preservation decisions and how these differences affect the emotional responses and decision‐making methods of men and women. A deep understanding of physiological and psychological factors can help us provide more targeted fertility preservation services and advice for men and women.

Sub‐sub‐theme 1.2.2 “Beyond biology: The role of cultural stereotypes in gendered perceptions of fertility”

By integrating related literature, we found that seven studies explicitly pointed out that culture has a significant impact on the attitudes and decisions of men and women when facing fertility issues.^34^, ^35^, ^36^, ^47^, ^50^, ^57^, ^58^ This is mainly reflected in the way culture shapes gender roles, forming stereotypes, and influencing patients' cognition and handling of fertility issues through moral norms. Besides, the influence of culture on the fertility concept is also significant.

Three studies indicate that the stereotype of men and women in the culture expects men to show a strong, brave, and decisive image,^34^, ^57^, ^58^ manifesting a quick and rational decision‐making approach to fertility issues. Conversely, another three studies show that women in culture are often seen as the primary bearers of the responsibility for pregnancy, childbirth, and raising children. This role expectation makes women pay more attention to their fertility and the undertaking of the mother role, possibly showing more emotional responses and hesitation.^34^, ^36^, ^47^

In addition, the shaping of the fertility concept by culture also affects family responsibility and inheritance. Three studies indicate that the culture generally expects women to conceive, give birth, and care for children, which is regarded as an important sign of women realizing their life value.^34^, ^36^, ^50^ In contrast, men's fertility responsibility is relatively light, paying more attention to career and family economic support. This may lead men to focus more on career and economic responsibilities when facing fertility issues, with relatively less attention to fertility. Therefore, during cancer treatment, men's fertility preservation issues are often downplayed, possibly leading to their fertility‐related psychological needs being overlooked or belittled.

“I would shy away about taking a guess on how it would affect a female, but I would imagine it would be harder, just because, you know, with women having babies, it's more, I guess, emotionally different for them to have children, so it might affect them psychologically more. One thing I've always thought is that if I can't have children, then even though it's obviously different, I would adopt. I guess that might be a little bit harder to do because the females actually carry the baby, and it might be different psychologically that way.” (P.60)^34^

“If I was a woman and there were questions about my fertility, I think I would be a lot more anxious. And I think that's because in our society, there's still much more duty placed on the woman to be able to provide a child. So, as a man, if I have a partner and we want to have kids a really viable option is to just get a sperm donor. And my partner still gets to go through pregnancy and have a child. But that's a lot; that's not the same at all if the gender role is reversed. So I think there would be more of a feeling of inferiority for a woman.” (P.61)^34^

The cultural shaping of fertility views and gender roles has profound implications. Men often prioritize career and financial responsibilities when facing fertility issues, while women might focus more on their ability to bear children. Hence, clinical professionals need to fully understand these cultural influences to better address the diverse needs of patients, especially when handling fertility concerns.

Sub‐sub‐theme 1.2.3 “Diverse focus points: Unpacking gender differences in fertility”

A total of 10 studies have demonstrated the differing focal points of men and women when dealing with fertility issues.^33^, ^35^, ^36^, ^46^, ^47^, ^49^, ^50^, ^52^, ^57^, ^58^ Seven of these studies show that, for female patients who become pregnant posttreatment, they endure both hope and distress simultaneously.^49^, ^50^ These patients are more concerned about their fertility and are more inclined to be fully involved in the treatment process. From the time of cancer diagnosis to the birth of a newborn, their focal point may shift gradually from their own health to the health of their child.^33^, ^36^, ^46^, ^47^ Their satisfaction with medical personnel's work is also related to whether the patient's fertility issues are considered throughout the process.^50^, ^52^ Institutions that take fertility issues into account throughout the treatment process and provide related care often receive higher satisfaction ratings.

In contrast, five studies suggest that male cancer patients may be more concerned about their ability to regain fertility.^36^, ^56^ While they also retain fertility, their actual concern for the state of fertility is relatively low. Even though they demonstrate enthusiasm for the evaluation and preservation of fertility prior to treatment,^57^, ^58^ and their satisfaction with fertility preservation tends to be higher,^57^ they often no longer pay attention to fertility testing after treatment.^33^

“Don't know what the effects have been and haven't been back to find out if I (Men) am sterile or not.” (P.8)^36^

These findings reveal the different focal points of men and women when faced with cancer and fertility issues, providing important information for health care providers on how to better meet the needs of cancer patients of different genders.

Sub‐sub‐theme 1.2.4 “Gender differences in managing fertility struggles”

A total of 14 studies support the subject of gender differences in managing fertility issues, sharing and explaining fertility problems, and satisfaction with fertility preservation.^33^, ^34^, ^35^, ^36^, ^46^, ^47^, ^48^, ^50^, ^51^, ^52^, ^53^, ^55^, ^56^, ^57^

Two studies reveal that women tend to be more open about their fertility issues and seek emotional support,^47^, ^55^ while men demonstrate greater reservation and selectivity in sharing these issues. In the total body of 14 studies, it is shown that women face more issues with fertility, requiring more complex information.^57^ Due to the complexity of their physiological structure and diversity of treatment methods, their fertility issues are often more affected by physiological cycles and diseases.^47^, ^51^, ^56^ Consequently, their comprehension when facing related information may be weaker,^34^, ^35^, ^50^, ^56^ and during the process of pregnancy and childbirth after treatment, they may continuously worry and be affected.^47^ Moreover, the complexity of the decision‐making process might hinder the usage of fertility protection technology, and might even lead to regret after decision‐making.^52^, ^56^ On the other hand, six studies indicate that men tend to exhibit smaller fertility concerns.^33^ Their understanding of information is sufficient to facilitate making rational fertility decisions.^17^ Particularly during sperm preservation, they are more concerned about the ability to restore fertility,^35^, ^56^, ^57^ and their satisfaction with fertility preservation decisions is high. Men may feel embarrassed when explaining fertility issues to others,^36^, ^57^ and are more inclined to share the emotional significance of fertility in their lives.^34^

Based on the aforementioned studies, we can draw the following conclusions for guidance in clinical practice: Significant differences exist between males and females in terms of dealing with fertility issues, sharing and explaining fertility problems, and satisfaction with fertility preservation. Women tend to be more open about their fertility issues and seek emotional support, while men demonstrate more restraint in sharing these issues. Furthermore, due to differences in physiological structures and treatment methods, women face more challenges in fertility issues and need more complex information. Therefore, women's comprehension when dealing with related information might be weaker, and they may continuously worry during the process of pregnancy and childbirth after treatment. Men, on the other hand, are more rational and composed when making fertility decisions, with their primary concern being the ability to restore fertility and high satisfaction with fertility preservation decisions. However, they may feel embarrassed when explaining fertility problems to others.

These findings provide important guidance for clinical practitioners when dealing with fertility issues in male and female patients, necessitating different communication methods and treatment strategies according to gender differences in order to better meet the needs of patients of different genders.

#### 3.4.2 Synthesized finding 2

Theme 2.*“The influence of Age: Experiences with fertility issues among cancer patients at different life stages.”*

Sub‐theme 2.1 “Peering into young minds: Perception and response to fertility issues among pediatric and adolescent cancer patients”

Supported by six studies, it is demonstrated that children and young cancer patients show unique cognitive and coping strategies when dealing with fertility issues.^33^, ^34^, ^35^, ^48^, ^52^, ^56^, ^57^ These individuals face changes in their physical condition that can greatly impact their social relationship development, particularly when these changes might affect future romantic relationships and fertility issues.^33^, ^34^, ^56^ Due to age restrictions, their communication, cognition, and decision‐making abilities may be weaker, thus necessitating additional guidance and support to understand complex fertility preservation issues.^33^, ^34^, ^52^, ^56^, ^57^ Their parents or guardians play a pivotal role in this process, especially in evaluating the impacts of treatment and potential fertility outcomes.^35^, ^56^ As for their coping mechanisms, some patients opt for positive strategies, such as accepting and making do, positively balancing reproductive concerns and loss of reproductive capacity, sharing with partners, and prioritizing “normality” over fertility concerns.^33^, ^35^, ^48^, ^56^ On the other hand, some resort to negative strategies, including desiring to postpone concerns, reliance on assisted reproductive technologies, and emotion‐focused coping.^33^, ^48^ Three studies suggest that communication with professionals can alleviate their fertility concerns, and the assistance of parents is also crucial.^35^, ^56^, ^57^

“But kids are a big deal in my community, because, like I said, people get married young and have kids and everything, so it's definitely an issue when it comes to dating. Like the guys, you can say, “I'm fine,” but they might want more proof than that. And I don't have proof right now, you know? One guy I was going to date … his family was like, they wanted me to take some sort of test.” (P.51)^33^

“So, an issue that [doctors] always talked about was infertility, but there is no 100% certainty. If I am not mistaken, the doctor spoke to my mother, because I was very young. I didn't understand anything. My mom told me when I was about 12.” (P.87)^35^

The research reveals that children and adolescent cancer patients exhibit unique cognitive and coping strategies when facing fertility issues. The physical changes they confront could potentially affect their future social relationships and fertility, making it necessary for them to receive additional guidance and support. Parents or guardians play a pivotal role in this process, providing crucial support and guidance in evaluating the impacts of treatments and possible fertility outcomes. Patients' coping strategies vary, with some choosing to respond positively and others negatively. Communication with professionals and support from parents can effectively help them deal with fertility issues.

These findings have significant implications for clinical practice. First, health care professionals need to understand and respect patients' cognition and coping strategies. Second, offering guidance and support about fertility issues to patients and their families is crucial. Moreover, engaging in effective communication with patients and their families to help them understand and cope with fertility issues is also highly important.

Sub‐theme 2.2. “Facing the challenge: Perception and strategies for handling fertility issues in cancer patients of other ages”

Twelve studies^33^, ^34^, ^43^, ^44^, ^45^, ^46^, ^47^, ^49^, ^50^, ^52^, ^57^, ^58^ have demonstrated a diverse and complex approach in cancer patients across different age groups to fertility issues. Concerns span multiple facets, such as personal fertility and parenting abilities,^43^, ^44^, ^46^, ^49^, ^58^ breastfeeding,^44^ the health of a potential fetus,^45^ the health of the patient themselves,^43^, ^45^ their children's health and childcare,^43^, ^44^ intimate relationships,^34^, ^43^ disease prognosis and survival,^57^ and aspects of sex life.^33^, ^34^ Accompanying these concerns are intricate emotions including guilt toward parents, spouse, and family,^44^ remorse and self‐blame,^45^ fear of death,^46^ and a sense of losing womanhood.^47^, ^50^, ^52^ Five studies^43^, ^46^, ^47^, ^48^, ^51^ assert the necessity of acknowledging and accepting potential fertility impairments, a journey often accompanied by periods of grief and anxiety, especially when discussing fertility concerns and expectations with partners.

Eight studies^43^, ^46^, ^47^, ^48^, ^51^ point to self‐regulation mechanisms adopted by patients, such as seeking medical support, facing anxiety squarely, adjusting mentality to overcome anxiety,^43^, ^48^ changing unhealthy lifestyle habits,^43^ enhancing self‐health awareness,^43^, ^47^ accepting reality,^33^, ^48^, ^56^ confronting worries,^55^ and sharing possible infertility risks with partners.^33^, ^34^

According to five studies,^45^, ^50^, ^52^, ^53^, ^54^ patients may seek help from professionals like doctors and psychotherapists to better comprehend and accept their potential fertility status. Decisions on fertility preservation measures such as freezing sperm or eggs are often influenced by factors like cost, time, and potential success rate, as suggested by six studies.

“Its been really hard not knowing [about fertility status]. I think when I first found out [about the risk of infertility] it was just horrible. And I didn't really deal with it, and I kind of just swept it under the rug, and then I started getting anxiety. Honestly, the worst part of cancer I think for me was the recover. this anxiety and fear, all that stuff that comes after, is almost worse… It's like such a process – such a grieving process.” (P.57)^34^

Seven studies^43^, ^44^, ^45^, ^46^, ^49^, ^50^, ^51^ elaborate on the struggle to balance treatment, fertility, and quality of life, which includes envisioning future lives, pondering the possibilities of having children (through surrogacy or adoption, for instance), and aligning fertility issues with other life goals and values. Adjusting expectations for romantic relationships, family, and community life is essential to adapting to potential fertility changes.^33^, ^46^, ^49^

Lastly, two studies^43^, ^53^ mention that patients often seek support within the community and health care system, encompassing fertility preservation services, psychological support, and financial aid. These resources can offer critical assistance when dealing with the challenges fertility issues pose, such as psychological stress, social pressure, and economic strain.

“It made me realize life is too short to be upset over petty issues and hold grudges, and that forgiveness is important to move forward in life. I realized that looking backward at past mistakes wasn't as important anymore. Being present and engaging with my family and telling them how important they are to me gives me the most meaning now.” (P.201)^47^

In conclusion, the literature reveals a complex and multifaceted response from cancer patients to fertility issues, spanning personal, emotional, and lifestyle concerns. Acknowledgment and acceptance of potential fertility impairments play a pivotal role, often involving periods of grief, anxiety, and difficult conversations with partners. Patients utilize various self‐regulation mechanisms to cope, ranging from seeking professional help to confronting anxieties directly, making lifestyle adjustments, and sharing potential fertility risks with their partners. Decision‐making around fertility preservation measures, often influenced by practical factors such as cost and potential success rates, presents another layer of complexity. Furthermore, balancing treatment, fertility, and quality of life emerges as a recurring theme, requiring adjustments to expectations for romantic relationships, family, and community life. Lastly, support from both the community and healthcare systems, including fertility preservation services, psychological support, and financial aid, is identified as a valuable resource to navigate these challenges. Future work should aim to further understand and support the diverse experiences and needs of cancer patients regarding fertility issues.

Sub‐theme 2.3. “The age gap: Differences in understanding and addressing fertility issues across age groups of cancer patients.”

Significant differences were discerned when comparing the concerns and behaviors regarding fertility across different age groups of cancer patients, with 14 studies corroborating this theme.^33^, ^34^, ^35^, ^43^, ^44^, ^45^, ^46^, ^47^, ^49^, ^50^, ^52^, ^56^, ^57^, ^58^ Four studies emphasized the unique challenges pediatric and adolescent cancer patients face due to their developmental stages.^33^, ^34^, ^35^, ^56^ Their concerns often lean more toward the impact of their illness on intimate relationships, such as dating and partner relationships, and how to communicate fertility‐related matters with future partners. This focus might not be as extensively considered in other age groups.

Furthermore, communication barriers may arise when discussing their fertility issues and expectations, as highlighted in two studies.^34^, ^35^ These barriers could be age‐related and often require family participation, creating potential tension between autonomy and family involvement, as indicated by another two studies.^52^, ^57^ These factors contribute to the call for more comprehensive support structures for this demographic.

For patients from other age groups who are contemplating or have already established their own families, they face a distinct set of challenges. Twelve studies jointly emphasize their primary concern: balancing the implications of their cancer treatments with their fertility and parenting capabilities.^33^, ^34^, ^43^, ^44^, ^45^, ^46^, ^47^, ^49^, ^50^, ^52^, ^57^, ^58^ Treatments like chemotherapy can indeed affect fertility, thereby creating a complex decision‐making environment.

“I think if she'd had told my parents that I couldn't conceive and not actually come to me, I think it'd have been worse. I'd have been I'd have been like, Well, why didn't she speak to me? How come you know and I don't? I'd have thought they were hiding more things from me. Where [the consultant] spoke to me more than she did my parents, she made sure we were all there together, not taking my parents away and speaking to them.” (P.384)^57^

In conclusion, understanding the divergent concerns and behaviors regarding fertility across different age groups of cancer patients is crucial. This underscores the need for a patient‐centric and age‐appropriate approach when providing care and support. Further research is required to elucidate the impact of these differences on quality of life and decision‐making processes, as well as how best to support patients coping with fertility concerns.

#### 3.4.3 Synthesized finding 3

Theme 3 *“The impact of treatment stages on fertility concerns: The evolution of perception and coping strategies in the course of cancer treatment”*

Sub‐theme 3.1. “Pretreatment: Navigating fertility ambiguities in the shadow of cancer”

Eight studies support this theme.^36^, ^42^, ^44^, ^45^, ^50^, ^55^, ^57^, ^58^ In the initial stages post‐diagnosis, patients may experience intense emotional reactions.^50^, ^57^ Several studies point out that a variety of negative emotions such as fear, anxiety, and uncertainty may arise due to the uncertainty of future fertility and the potential impact on fertility of treatment.^36^, ^44^, ^45^, ^50^, ^55^ At this time, patients need to make fertility preservation decisions in a short period of time.^50^, ^54^, ^55^ This process, filled with stress and confusion, further exacerbates patients concerns about future fertility, adding immense emotional pressure. Six studies all state that patients require in‐depth and comprehensive information^1^, ^3^, ^4^, ^12^, ^18^, ^21^ to understand the various impacts cancer treatment may have on their fertility, including the extent of the potential impact on their fertility by treatment, the fertility preservation measures that need to be taken, and how to balance their fertility needs while undergoing treatment. Four studies indicate that in‐depth discussions with medical professionals,^45^, ^55^, ^57^, ^58^ including doctors, nurses, counsellors, or reproductive medicine experts, etc., are essential for understanding as many potential options as possible, as well as the pros and cons of each option, potential risks, and long‐term impacts.

“When asked about it [fertility and pregnancy] at diagnosis, I didn't fully understand my options and felt pressured to make a quick decision with no information in front of me. Every day since my diagnosis, I've felt very upset that I didn't have more information at the time to make a more informed decision. Knowing that I will never be able to give birth because of the chemo has made it hard to accept.” (P.201)^47^

The studies predominantly corroborate that in the initial stages post‐diagnosis, patients experience intense emotions due to uncertainties related to future fertility and the impact of treatment. They are required to make fertility preservation decisions within a brief period, during which they need comprehensive information to understand the potential impacts of cancer treatment on fertility. Furthermore, in‐depth discussions with medical professionals are vital for understanding the potential options, their pros and cons, and their long‐term impacts.

Sub‐theme 3.2. “During treatment: Seeking fertility pathways amidst the whirlpool of life”

Seven studies underpin this theme.^36^, ^44^, ^47^, ^50^, ^51^, ^55^, ^56^ Distilled from two studies is the insight that during the treatment process, patients begin to experience some side effects of cancer treatment. Cancer patients may undergo physiological changes such as a decline in bodily function, changes in body shape, and reduced libido.^47^, ^51^ These changes may alter their fertility intentions and deepen their concerns about their fertility capacity. At this time, patients need to employ various coping strategies. Three studies indicate that these strategies may include adjusting their expectations,^44^, ^50^, ^55^ learning new stress management strategies, and seeking psychological support, such as counseling or participating in support groups.^50^, ^55^ As the treatment progresses, four studies find that patients may have updated needs for information about fertility preservation efficacy, opportunities for posttreatment fertility, and related issues.^36^, ^44^, ^55^, ^56^

“I was oestrogen positive, so I can't have any more children, which I desperately wanted; it's too much risk, and also, my ovaries stopped producing eggs on my 4th round of chemotherapy.” ^51^

“I think they should be in contact throughout your whole chemotherapy treatment. Once you stop having to go there, then you kind of feel a little bit lost and wonder, Now how do I get back to normal? It's almost like they give too much support at the start, and you get this expectation about what support you're going to receive, and then all of a sudden you don't see them at all.” (P.61)^53^

The literature broadly supports the idea that cancer treatment can induce various physiological changes, leading to alterations in fertility intentions and concerns about fertility capacity. To cope with these changes, patients may need to adjust their expectations, learn new stress management strategies, and seek psychological support. There is also an emerging need for updated information on fertility preservation efficacy and opportunities for posttreatment fertility as the treatment progresses.

Sub‐theme 3.3. “Posttreatment: Exploring the future of fertility amid hope and concern.”

Six studies substantiate this subject matter.^33^, ^43^, ^46^, ^49^, ^53^, ^54^ Three studies noted that following treatment, patients may harbor hope for the restoration of their fertility, especially those desiring pregnancy. They may feel excitement about future fertility plans but also possibly deep concerns due to declining fertility, health risks to offspring, and other issues.^43^ Balancing hope and distress becomes a significant task.^33^, ^46^, ^49^

Patients may need to understand whether their fertility has been restored and when they can attempt to conceive. Four studies mentioned the need for clear guidance on how to achieve fertility goals while recovering from cancer. These include the readiness to attempt pregnancy and the potential need for additional medical monitoring and support. Two studies focused on the needs of women who are either in the process of gestation or planning for it. They may proactively seek guidance from the medical team to ensure the health of themselves and their future child.^33^, ^53^ Two other studies emphasized that cancer survivors who are pregnant or planning to be may require additional support and attention. They may face various challenges during pregnancy, such as fatigue, nausea, weight gain,^49^ and worries about their disease affecting the health of their child.^46^, ^49^

“Do I need some advice from my doctor on whether I am suitable to have a baby or not according to my condition? What are the risks during pregnancy or delivery? I had a lot of complications during pregnancy, for example,don't they have a lot of complications now? Wouldn't I be at greater risk for some of the symptoms of pregnancy? Will having a child have an effect on my condition?” (P.29)^43^

“What you need to consult with your doctor may be how much and what medication to take, when and how to prepare for pregnancy, whether the indicators of pregnancy are normal, and whether there are any problems with the development of the fetus.” (P.197)^45^

Three studies discussed coping strategies, suggesting that patients might seek more medical advice.^46^, ^54^ This could include expert opinions on when they can attempt pregnancy and the type of medical support needed to achieve their fertility goals. They may also seek psychological support,^53^ including counseling to help them cope with the stress and anxiety associated with fertility.

Overall, the management of fertility issues by cancer survivors in the posttreatment stage requires support on multiple levels, including medical, psychological, and social. This support needs to respect and understand the needs and experiences of patients to help them find their own path in the complex emotional and decision‐making process.

### 3.5 ConQual summary

Table 2 presents the ConQual evaluation of the synthesized outcomes. The dependability of all synthesized outcomes was reduced owing to issues with the methodological quality. The credibility of the three synthesized outcomes decreased due to a combination of unequivocal and credible results. As a result, all findings were classified as low‐level evidence.

**TABLE 2:** ConQual summary of the findings.

Synthesized findings	Dependability	Credibility	ConQual score
“Gender differences in fertility concerns among cancer patients: Perspectives from men and women”	Downgrade 1 level^a^	Downgrade 1 level^b^	Low
“The influence of age: Experiences with fertility issues among cancer patients at different life stages”	Downgrade 1 level^a^	Downgrade 1 level^b^	Low
“The impact of treatment stages on fertility concerns: The evolution of perception and coping strategies in the course of cancer treatment”	Downgrade 1 level^a^	Downgrade 1 level^b^	Low

^a^ Due to recurring reliability issues across the primary studies included (most studies did not provide a statement positioning the researcher culturally or theoretically, and the researcher's influence on the research was not acknowledged),

^b^ One level was downgraded. Similarly, a mix of unequivocal and credible findings caused a further one‐level downgrade.

## 4 DISCUSSION

This systematic review and meta‐synthesis provide a comprehensive understanding of the inner experiences of cancer patients' fertility concerns from various perspectives. We have distilled three main integrative themes from 21 literature sources. The results show that different patients perceive fertility concerns, needs, and coping strategies differently when facing fertility issues. This includes differences by gender, age, and treatment stage.

Male cancer patients are more focused on the restoration of their fertility capabilities and the impact of treatment on future fertility.^33^, ^46^, ^47^ They hold a more rational attitude toward fertility preservation decisions and care more about the actual possibility of conception rather than emotional factors.^33^, ^34^, ^35^, ^57^ Although they need to receive professional advice on fertility issues, they may prefer to learn this information before starting treatment.^36^, ^49^, ^51^ In contrast, female cancer patients are more concerned about whether their fertility issues are adequately considered during treatment and the health of their children after treatment.^49^, ^52^, ^57^ When dealing with fertility issues, females tend to seek emotional support.^47^, ^55^ In fertility preservation decisions, females might be more emotionally influenced, with decisions often more affected by personal feelings and expectations.^33^, ^34^, ^35^, ^57^ They emphasize the importance of fertility, cherish femininity, and hope to continuously receive information and support about fertility issues throughout the treatment process.^36^, ^49^, ^51^ Some patients suggest that structured dialogue with patients before treatment and interdisciplinary cooperation may better address these concerns. Researchers have pointed out that the establishment of fertility care organizations, multidisciplinary teams, and oncological reproductive referral services can provide patients with more information support. With informed consent and autonomy, meeting the personalized needs of patients can reduce fertility anxiety levels.^26^, ^59^ In the future, more in‐depth research can be conducted according to the characteristics of each gender, while exploring other factors that may influence patients' attitudes, needs, and coping strategies regarding fertility concerns, such as age, educational background, and economic conditions. Medical service providers need to understand and respect these differences to better meet the fertility preservation needs of cancer patients.

In addition, this study summarized the gender differences in different experiences, needs, and coping strategies regarding fertility issues among men and women, providing an important theoretical basis for more detailed, personalized care needs, and improving fertility preservation services for cancer patients. First, physiological differences between men and women lead to differences in fertility concerns. The integrated results show that compared to men, women are more likely to be affected by physiological cycles, making the fertility preservation process more complex, increasing the difficulty of understanding for women, and even causing patients to change their treatment plans,^47^, ^51^, ^56^ regretting their decisions. Men can generally store sperm in a sperm bank, and the understanding of this process is relatively easier, overall showing higher satisfaction.^57^ The study shows that fertility preservation can alleviate fertility concerns among cancer patients.^60^ However, most participants (62.2%) have a low level of understanding of fertility preservation, or despite a positive attitude, “prioritizing cancer treatment,” “cancer recurrence due to fertility preservation,” and “the health of the next generation” can also affect the decision on fertility preservation.^61^ Cultural stereotypes shape gender and interact through moral norms affecting men's and women's cognition and handling of fertility issues. Cultural shaping of fertility concepts involves family responsibility and inheritance methods, which leads to female patients' emphasis on female fertility and maternal responsibilities after a cancer diagnosis. Clinical professionals need to fully understand these cultural influences to better meet the different needs of patients. There are significant differences in the focal points of fertility issues between men and women. Men are more concerned about whether fertility can be restored, while women are more concerned about whether their fertility issues were considered during treatment, as well as the health of their children after treatment.^33^, ^36^, ^57^ These differences may stem from biological sex differences and societal cultural stereotypes about gender roles.^34^, ^57^, ^58^ In clinical practice, medical service providers need to understand and respect these differences to better meet the needs of patients of different genders. In addition, there are significant differences in the strategies of men and women in dealing with fertility issues. Women tend to seek emotional support when facing fertility issues, while men show more contradictions and hesitations.^47^, ^55^ Studies by Drizin^62^ and Young^63^ found that fertility consultations before treatment are related to higher fertility anxiety. Medical staff should communicate with patients in a timely manner maintain communication, thereby alleviating fertility concerns due to a lack of knowledge in cancer patients. Medical staff should also actively accept relevant training to improve their knowledge level in the field of oncological fertility. Therefore, medical service providers need to understand these differences and provide corresponding support and resources to help patients better deal with fertility issues. Our research also found that men and women have different attitudes toward fertility preservation decisions. Men generally have a more rational attitude toward fertility preservation decisions, while women may be more influenced by emotions.^33^, ^35^, ^57^ These findings suggest that when providing fertility preservation suggestions to patients, their gender, emotions, and understanding ability need to be taken into consideration.

Adolescent and child cancer patients display unique characteristics in their understanding of and coping mechanisms for fertility issues. Due to age restrictions, adolescent and child cancer patients require extra guidance and support to comprehend the complexities of fertility preservation. However, the involvement of family members poses additional challenges.^35^, ^56^ Therefore, when health care professionals work with patients at different ages, they must understand and respect the patients' cognizance and coping strategies. They should provide guidance and support about fertility issues for the patients and their families, and communicate effectively to help them understand and deal with fertility issues.^33^, ^52^, ^56^ Adult cancer patients exhibit a variety of complex strategies when dealing with fertility issues. Their concerns span several areas, including personal fertility and parenting capabilities, breastfeeding, the health of potential offspring, their own health, their children's health and care, intimate relationships, disease prognosis and survival, as well as various aspects of sexual life. To address these issues, patients have adopted various self‐regulation mechanisms, such as seeking medical support, confronting anxiety, changing unhealthy lifestyles, enhancing self‐health consciousness, accepting reality, facing worries, and sharing potential infertility risks with partners, among others.^48^, ^51^ Balancing considerations of treatment, fertility, and quality of life at this stage is necessary. This includes envisioning future life, considering the possibility of having children (such as through surrogacy or adoption), and aligning fertility issues with other life goals and values. Adjusting expectations of romantic relationships, family, and community life to adapt to potential fertility changes is crucial.^33^, ^46^, ^49^ Adolescents and children with cancer, due to their developmental stage, face unique challenges such as focusing on the impact of the disease on intimate relationships and how to communicate fertility issues with future partners. For those who have already established their families or are considering doing so, the challenges they face are different. They mainly focus on how to balance the impact of their cancer treatment on their fertility and parenting abilities.^44^, ^49^, ^52^ Understanding the differing focuses and behaviors regarding fertility issues among cancer patients at different stages of life is crucial. This highlights the need for patient‐centered and age‐appropriate approaches when providing care and support. Further research is needed to elucidate the impact of these differences on quality of life and decision‐making processes, and how to best support patients dealing with fertility issues.

Cancer patients also exhibit differences in cognition, needs, and coping strategies for fertility concerns at different stages of treatment. Our integrated research results show that in the pretreatment stage, due to the uncertainty of future fertility and the potential impact of treatment on fertility, patients may experience a range of negative emotions, such as fear, anxiety, and uncertainty.^44^, ^45^, ^50^, ^55^ Patients need to make decisions about fertility preservation in a short amount of time, and they need in‐depth and comprehensive information to understand the potential impact of cancer treatment on their fertility.^42^, ^45^, ^58^ Other research results also show that fertility issues in cancer patients can have a variety of impacts. Patients' understanding of the timing of their own disease treatment, chances of survival, and whether it will affect the health of their offspring, doubts about the maturity of the technology, the reliability of sources of information on reproductive insurance, the scope of medical insurance coverage, the impact of anticancer treatment regimens on fertility, etc. can all affect the patient's decision‐making process, placing additional psychological pressure on the patient during the treatment process.^64^ Therefore, communication between the doctor and the patient about fertility issues is very important at this time. During treatment, cancer treatment may trigger various physiological changes, which may change their desire to have children and deepen their worries about their fertility.^47^, ^51^ To cope with these changes, patients may need to adjust their expectations, learn new stress management strategies, and seek psychological support.^44^, ^50^, ^55^ Therefore, clinical medical staff should strengthen education about various fertility preservation techniques and their application scope and timing, scientific contraception during anticancer treatment, and the risk and subsequent handling of accidental pregnancies.^65^ After treatment, patients may hope to restore fertility, especially those who wish to become pregnant. They may be excited about future fertility plans, but they may also be deeply worried about issues such as decreased fertility and concerns about the health of their children.^43^, ^46^, ^53^ At this stage, patients may need medical advice,^46^, ^54^ including expert opinions, to understand when they can try to get pregnant and what type of medical support they need to achieve their fertility goals. Overall, our research provides a deep understanding of the impact of the stages of cancer treatment on fertility concerns and possible coping strategies. This will help medical teams to provide more effective fertility support and guidance for cancer patients. These findings emphasize the importance of understanding patients' needs and experiences during cancer treatment, and helping them find their own path in the complex emotional and decision‐making process.

Lastly, we would like to emphasize that our robust study results are the product of a rigorous data analysis process that includes meticulous data extraction, thematic analysis, and cross‐validation to ensure accuracy and reliability. Furthermore, strict adherence to recognized qualitative research standards has heightened the effectiveness of our interpretations. Through result triangulation and peer review, our intention has been to mitigate researcher bias and enhance the credibility of our findings. However, it is important to acknowledge that our study is not without limitations. Despite our sampling strategy aiming to maintain diversity, discrepancies in sample sizes among different categories may introduce bias. Hence, considering larger and more balanced samples in future research endeavors would help alleviate this concern. Additionally, our research predominantly focuses on qualitative aspects, yet the integration of quantitative data may offer a more comprehensive understanding.

### 4.1 Study limitations

Our study has several limitations. First, there is an uneven gender distribution of research participants. Most of the literature included in this study pertains to female cancer patients, with relatively less information on male cancer patients. This may limit our understanding of fertility concerns among male cancer patients. Second, potential bias in age grouping, The age division of the study was determined based on whether a specific age group was mentioned in the title. However, this method may lead to some inaccuracies in age grouping.

Besides, data collection methods may affect the quality of the study. The study includes some studies that collected data on open‐ended questions using a letter‐writing method, but this method might be affected by response rates and the quality of language expression.

Last, subjectivity may exist in the division of treatment stages. In this study, we divided the study into stages before, during, and after treatment. However, such division might be subjective, potentially affecting our understanding of the changes in fertility concerns among cancer patients. In summary, the synthesized findings of this study were limited by the quality and reporting of the included studies. Furthermore, the meta‐aggregation approach does not seek to reinterpret the implications of original findings. Therefore, the credibility of the synthesized findings of this study was limited by the reporting of the included studies. Additionally, the quality and validity of the study are influenced by the studies included, while the meta‐aggregation method underscores the avoidance of reinterpreting the meaning of the original research findings. These factors collectively influence the conclusions and insights derived from our study.

### 4.2 Clinical implications

From the perspective of the research itself, our study carries significance in several ways. First, we delved deeply into the psychological experiences of cancer patients facing fertility issues, using gender as a classification. Our findings reveal significant differences in the needs and coping strategies of male and female patients when dealing with fertility issues. This provides a basis for doctors to offer more personalized medical services when addressing fertility issues in patients. For instance, doctors could provide more information and support about fertility capabilities and posttreatment pregnancy for female patients. For male patients, emphasis could be placed on explaining and providing advice on the restoration of reproductive abilities and relevant treatment options. Second, we categorized cancer patients according to age and discovered significant differences in the understanding and focal points of fertility issues among patients of different age groups. This reminds us to fully consider the influence of age on patients' understanding and decision‐making regarding fertility issues in order to offer more effective assistance. For example, we might need to involve parents or other family members to help adolescent patients better understand and handle fertility issues. For adult patients, more psychological support and fertility‐related information might be needed. Moreover, we studied the psychological experiences of cancer patients at different treatment stages, finding distinct variations in their concerns and coping mechanisms when dealing with fertility issues. This highlights the need for personalized services at different treatment stages to better meet their needs. For instance, before treatment, we could offer more decision‐making support to help patients understand and deal with potential fertility issues. During treatment, psychological support could be provided to help them cope with physical and physiological changes. After treatment, more information and support regarding pregnancy and childbirth could be given. Additionally, our research could assist health care providers in better understanding the needs and coping strategies of cancer patients facing fertility issues, thereby developing more targeted educational materials and programs to help patients better understand and manage their fertility issues. This study also lays the foundation for future research in related fields. For example, future research could further explore the psychological experiences and coping strategies of cancer patients of different genders, ages, and treatment stages regarding fertility issues. By better understanding and supporting cancer patients in handling fertility issues, their quality of life can be improved.

In summary, our research results emphasize the importance of personalized medical services when dealing with fertility issues in cancer patients. This is crucial to improving the handling of fertility issues in cancer patients and enhancing their quality of life. This not only conforms to international paper‐writing standards but also provides practical references for clinical doctors.

## 5 CONCLUSIONS

Through a comprehensive meta‐analysis, this study delves into the psychological experiences of cancer patients facing fertility concerns. We meticulously dissect the attitudes, needs, and coping mechanisms toward fertility issues from the perspectives of different genders, age groups, and stages of cancer treatment. This innovative approach not only facilitates a more profound understanding of the complex emotions and needs of cancer patients when dealing with fertility problems but also provides fresh insights on how to deliver more effective medical assistance, psychological counseling, and relevant informational services.

## AUTHOR CONTRIBUTIONS

**Ying Dong:** Conceptualization (equal); data curation (equal); formal analysis (equal); investigation (equal); methodology (equal); project administration (equal); resources (equal); software (equal); supervision (equal); validation (equal); visualization (equal); writing – original draft (equal); writing – review and editing (equal). **Zhenyu Yue:** Data curation (lead); formal analysis (lead); methodology (lead); resources (lead); supervision (lead); writing – original draft (lead); writing – review and editing (supporting). **Huan Zhuang:** Conceptualization (supporting); formal analysis (supporting); methodology (supporting); writing – review and editing (supporting). **Chen Zhang:** Data curation (supporting); formal analysis (supporting); methodology (supporting); supervision (supporting). **Yu Fang:** Data curation (supporting); formal analysis (supporting); methodology (supporting); supervision (supporting). **Guichun Jiang:** Methodology (supporting); supervision (supporting).

## CONFLICT OF INTEREST STATEMENT

The authors report no real or perceived interests related to this article that can be construed as a conflict of interest.

## ETHICAL APPROVAL

This article does not contain any studies with human participants or animals performed by any of the authors.

## Data Availability

The data that support the findings of this study are available from the corresponding author.

## APPENDIX A

**TABLE A1:** Characteristics of the studies.

No	Study (year), Country	Design (date collection method)	Inclusion criteria: treatment period	Phenomenon of interest	Recruitment and participants	Main findings
1	Xiao et al (2023), China^42^	Descriptive phenomenological approach (semi‐structured interviews)	Receiving systemic intravenous chemotherapy/endocrine therapy	To deeply understand the current experience of fertility information support for young breast cancer patients and to provide further evidence supporting the development of a fertility information support project	18 young breast cancer patients (19–39) were selected from a hospital in Hunan Province	Three themes were identified: (1) information anxiety; (2) reproductive concerns; and (3) family support.
2	Wang Bin (2022), China^43^	Descriptive phenomenological approach (semi‐structured interviews)	1–4 years after surgery	To explore the real experience of productive concerns in young patients with cervical cancer who receive fertility‐sparing surgery, To understand in depth the strategies and support needs for coping with reproductive concerns during the course of the disease and the need for fertility protection during treatment	17 young patients (20–40) with cervical cancer who receive fertility‐sparing surgery from a tertiary level A oncology specialty hospital	Three themes were identified: (1) multiple manifestations caused by reproductive concerns; (2) guilt feelings caused by illness and fertility problems: guilt to parents, guilt to family; (3) need for more health support: health care information, family support, social support; and (4) strategies to actively deal with psychological problems caused by reproductive worries.
3	Xiao (2021) China,^44^	Descriptive phenomenological approach (depth interviews)	Patients treated in hospital**s**	To explore the current status and real psychological experience of fertility concerns among women with breast cancer in their childbearing age	12 breast cancer patients (18–40) of childbearing age in the Department of Breast Oncology	Four themes were identified: (1) self‐concern; (2) concerns about the health and care of children; (3) information support for childbirth; and (4) negative emotional experiences as a result of not having a child.
4	Tang (2020), China^45^	Descriptive phenomenological approach (semi‐structured deep interviews)	Inpatient postoperative patients	To explore the concerns of young females with thyroid cancer through in‐depth interviews so as to provide a basis for formulating the content of reproductive knowledge	13 young female thyroid cancer patients (18–39) admitted after surgery to Jiangsu Cancer Hospital	Three themes were identified: (1) worry and remorse; (2) desire for communication and support; and (3) needs for scientific reproductive knowledge.
5	Azizi et al (2023), Iran^46^	Qualitative descriptive study (semi‐structured interviews)	Female breast cancer patients who have completed treatment and experienced pregnancy	The present study is a mixed‐methods study conducted in three phases. In Phase 1, the researchers performed a qualitative content analysis study to explore the perceived needs and experiences of pregnancy among Iranian BC survivors	Participants were 16 BC survivors referred to two specialized and sub‐specialized clinics and hospitals in Sari, Northern Iran	Six themes were identified: (1) pregnancy and childbirth problems,” (2) maternal mental health problems,” (3) “social problems; (4) “marital instability,” (5) need to support,” and (6) “need to resort to spirituality,”
6	Carr (2022) US^47^	Qualitative descriptive study A seven‐question online survey (open‐ended questions)	Receiving breast cancer treatment	The current study explored the impact of cancer‐related fertility concerns on existential distress and meaning‐making among female breast cancer (BC) patients of childbearing age and assessed support needs	98 Recruitment was conducted via a review of medical charts for patients (18–45) seen in the oncology breast clinic and via recruitment flyers distributed through email (LISTSERVS)	Two themes were identified: (1) BC patient sources of existential distress from cancer‐related fertility concerns; and (2) meaning‐making with fertility‐related existential concerns.
7	Benedict (2016), US^33^	Grounded theory (semi‐structured individual interviews and focus groups)	Completion of anticancer treatment for at least 6 months	To explore AYAs' discussions of fertility in the context of discussing their survivorship experiences	Individual interview (*n* = 26) and a focus group (four groups, *n* = 17). Recruitment letters were sent to 90 survivors (16–24) and/or parents with follow‐up phone calls.	Three themes were identified: (1) fertility concerns; (2) emotions raised when discussing fertility; and (3) strategies used to manage fertility concerns.
8	Quinn (2013), US^48^	Qualitative descriptive study (cognitive debriefing interview)	Before diagnosis	The purpose of this qualitative study was to assess the coping styles of female adolescent cancer patients regarding the potential loss of fertility	14 participants (12–18) were recruited from two pediatric cancer centers	Two themes were identified: (1) emotion‐focused coping; and (2) problem‐focused (engagement) coping.
9	Vanstone (2021), Canada^49^	Grounded theory (semi‐structured, one‐on‐one interview either over telephone or video conferencing (Zoom))	Currently in the perinatal period	To better understand women's experiences of pregnancy and the postpartum period following cancer treatment through a qualitative analysis	10 women (<45) were recruited from Sunnybrook Health Sciences Centre in Toronto, as well as through online cancer support platforms and social media sites	Four themes were identified: (1) revealing how women go through a process of grief related to potential fertility loss; (2) conditional joy during and after pregnancy due to the lingering weight of cancer; (3) frustration with a lack of resources regarding perinatal health after cancer; and (4) hope as they enter into motherhood.
10	Newton (2021), Canada^34^	Qualitative descriptive study (in‐depth interviews)	Have had no cancer treatments within the 2 years prior to the study interview	To describe the challenges young adult childhood cancer survivors (ACCS) experience living with an unknown fertility status	25 ACCS (15 females, 10 males) (19–36) were recruited for a larger study examining ACCS narratives of health management through existing follow‐up clinics at both the children's and adult cancer centers, as well as online via specific forums and websites	Three themes were identified: (1) the marked psychological burden, which included fear, worry, anxiety, and sadness, was ubiquitous; (2) romantic relationships were negatively affected and entailed fear of disappointing one's partner, the difficulty of discussing fertility issues, and shying away from romantic relationships; (3) communication challenges with health care providers were apparent across the cancer trajectory, made worse by provider knowledge gaps and dismissal of fertility concerns; and (4) well‐known gender stereotypes about pregnancy and childrearing were replicated, while the emotional and life impacts that uncertain fertility could exert on males were minimized.
11	Jardim (2021), Brazil^35^	Qualitative descriptive study (semi‐structured interviews)	Having completed cancer treatment for 5 years and a disease‐free status	To uncover fertility‐related concerns and uncertainties in adolescent and young adult (AYA) childhood cancer survivors	24 participants (18–24) were recruited from an onco‐hematology outpatient clinic at a university hospital in Brazil	Four themes were identified: (1) knowledge about fertility; (2) emotional impact and fertility‐related uncertainty; (3) sharing the possible risk of infertility with partners; and (4) the need for information on possible loss of fertility.
12	Wang, (2020), Australia^50^	Qualitative descriptive study (semi‐structured telephone interviews)	At the time of their cancer diagnosis	To explore the fertility‐care experiences and reproductive concerns of reproductive‐age cancer patients at the time of their cancer diagnosis who have had access to onco‐fertility care	9 male patients and 21 female patients (15–44) were recruited from multiple hospital sites across Australia and New Zealand via the Australasian Onco‐fertility Registry	Five themes were identified: (1) satisfaction with onco‐fertility care; (2) a need for individualized treatment and support; (3) desire for parenthood; (4) fertility treatment can be challenging; and (5) fertility preservation provides a safety net for the future.
13	Ussher (2018), Australian^36^	Qualitative descriptive study (in‐depth one‐on‐one interviews)	After cancer diagnosis and treatment	To gain insight into the gendered experience of cancer‐related fertility concerns across age groups, cancer types, and parity	61 women and 17 men Participants (18–58) responded to advertisements circulated across Australia through social media, media stories in local press, advertisements in cancer and caregiver‐specific newsletters, cancer support groups, hospital clinics, and local Cancer Council websites and telephone helplines	One theme was identified: a threat of biographical disruption, which impacted life course and identity for both women and men.
14	Perz (2014), Australia^51^	Qualitative descriptive study (open‐ended responses)	From cancer diagnosis to survival	This qualitative study examines the subjective experience of infertility among a large sample of Australian women with breast cancer	381 participants were drawn from the membership of a national organization for Australians affected by breast cancer	Five themes were identified: (1) negative responses to infertility and early menopause; (2) sexual changes associated with menopause and infertility; (3) uncertainty and anxiety about fertility status; (4) information and fertility preservation; and (5) acceptance of the end of fertility.
15	Dryden (2014), Australia^52^	Constructivist qualitative study (semi‐structured one‐to‐one interviews)	From cancer diagnosis to survival	To explore younger women's construction of their fertility post‐cancer and their discussions of fertility with health care professionals from a social constructionist epistemology	Participants (18–26) responded to advertisements circulated nationally through cancer support groups, media stories in local press, advertisements in cancer and caregiver‐specific newsletters, hospital clinics, and local Cancer Council websites and telephone helplines	Two themes were identified: (1) The construction and experience of fertility in the context of cancer; and (2) Constructions of interactions with health professionals.
16	Kirkman (2013), Australia^53^	Qualitative descriptive study (in‐depth qualitative interviews)	At least 1 year post‐diagnosis	To learn from women about their experiences of cancer care in relation to their fertility, to attend to their recommendations to clinicians, and ultimately to inform and enhance the provision of supportive care to these women	10 women breast cancer patients (26–45) were sought in collaboration with BreaCan, a statewide service in the State of Victoria, Australia	Four themes were identified: (1) Fertility is important to women diagnosed with breast cancer; (2) Women must make rapid decisions under stressful circumstances; (3) Women experience varied care; and (4) Multidisciplinary care is valued.
17	Penrose (2012), Australia^54^	Qualitative descriptive study (face‐to face interview)	At least 1 month posttreatment	To assess the fertility concerns among cancer survivors aged 50 and under as part of a larger study investigating the survivors' concerns regarding fertility, sexuality, and parenting	25 patients (19 women) (27–50) were identified from an oncology database and invited to participate via letter or during a clinic consultation	Four themes were identified: (1) Fertility represents more than child‐bearing capacity; potential fertility loss was a concern for participants, irrespective of their desire for future children; (2) Assumed infertility: There was a tendency for participants to assume that they were infertile; (3) Lack of information regarding decision‐making and fertility: The respondents reported a perceived lack of consideration of fertility at diagnosis by medical professionals, and this impacted upon the decision‐making process; and (4) Participant recommendations: The respondents wanted more information and for support services to be offered.
18	Corney (2014), UK^55^	Qualitative descriptive study (individual semi‐structured interview)	At least 6 months after diagnosis	The aim of this qualitative study was to investigate in detail the fertility‐related experiences of young childless women with breast cancer, including the information they received, the fertility preservation options given, and the dilemmas they faced	19 childless women (<45) were recruited by contacting the breast cancer charities.	Nine themes were identified: (1) when fertility was first discussed; (2) options given on fertility preservation prior to chemotherapy; (3) those not given the choice or cautioned against it; (4) those not given chemotherapy; (5) fertility information and advice on pregnancy post‐chemotherapy and radiotherapy; (6) dilemmas and concerns; (7) worries over what the future might hold; (8) childless single women; and (9) recommendations made by the women.
19	Crawshaw (2010), UK^56^	Exploratory qualitative study (in‐depth single interviews)	From cancer diagnosis to survival	This study reports on its hitherto unreported impact over the time from diagnosis to survivorship	38 participants (<30) were recruited between 2004 and 2006 by senior doctors at routine appointments at three English regional pediatric oncology units	Four themes were identified: (1) prioritizing “normality” and marginalizing fertility; (2) fertility concerns compromising “normality;” (3) ongoing impairments or health concerns mediating fertility matters; and (4) fertility concerns dominating the cancer legacy.
20	Crawshaw (2009), UK^57^	Grounded theory study (in‐depth single interviews)	Not receiving treatment at the time of being approached about participation in this research	This study aims to provide the first report of male and female adolescent cancer patients' experiences of having fertility and associated decision‐making matters raised at diagnosis	38 male and female (16–30) survivors were recruited through three regional pediatric oncology units in the North of England	Seven themes were identified: (1) level of prior knowledge about cancer‐related infertility; (2) impact of being told about fertility matters at around diagnosis (adverse reactions, less pronounced reactions); (3) views about having fertility raised (general, female‐specific views); (4) decision‐making about fertility preservation (men); (5) age at diagnosis; (6) parental and family involvement; and (7) views on information received (men, women).
21	Chapple (2007), UK^58^	Qualitative interpretive approach (Narrative interviews)	Diagnosed with cancer, they had either recovered from cancer, were in remission, or were still being treated	To explore fertility issues for young men who had been diagnosed and treated for cancer and to examine communication problems surrounding these fertility issues	21 young men (16–26) previously treated for cancer in the United Kingdom	Four themes were identified: (1) the importance of choice; (2) the need for more counseling; (3) concerns about sperm banking; and (4) feelings about possible infertility.

**TABLE A2:** Synthesized findings.

Findings	Categories	Synthesized findings
Men: Attitude: (Vary over time,^21^ fear of losing male identity,^13^ embarrassment when discussing fertility,^21^ hopeful about fertility preservation, minimal fertility‐related distress)^7^, ^13^, ^21^ Demands: fertility recovery,^19^ more counseling^21^ Response method:performing sperm storage,^20^ communicating with others^10^	"Dual perspectives: unifying the distinctive experiences of men and women in facing fertility issues"	"Gender differences in fertility concerns among cancer patients: perspectives from men and women"
Women: Attitude: Negative emotional experiences (anxiety, self‐doubt, regret, self‐blame, and stress, guilt, dating and partner relationships, health risks, life narrative,);^1^, ^3^, ^4^, ^5^, ^8^ impact of infertility on life course and identity;^7^, ^10^, ^13^ multiple manifestations caused by fertility (body and feminine identity, such as hair loss, weight fluctuation, and premature aging),^5^, ^13^, ^14^, ^15^ fertility is important;^16^, ^17^ lack of information regarding decision‐making and fertility^1^, ^4^, ^14^, ^17^. Demands: Personal (disease prognosis and survival, fertility and breastfeeding, sex life, fertility preservation decisions, spirituality, treatment and support);^3^, ^5^, ^12^, ^17^ information support (fertility technology protection, the timing of pregnancy and safety of childbirth);^1^, ^3^, ^17^ desire for communication and support, multiple sources of scientific reproductive knowledge^4^, ^10^, ^16^ Response Method: Accept reality;^8^, ^14^ seek support (medical, information, family and social);^1^, ^2^, ^12^ change bad lifestyle;^2^, ^8^ distress;^8^, ^14^, ^19^ meaning making;^6^, ^8^ having hope while suffering;^7^, ^19^ women's advice to health care providers^12^, ^16^.		
*"Biology dictates: unraveling gender differences in fertility* 7, 10, 11, 13 Men are more feasible in the onco‐hematology setting^11^, ^21^ Males were more likely than females to indicate minimal fertility‐related distress^7^, ^11^, ^13^ Women face more complexity in fertility preservation decisions , including time pressure, emotional effects, managing early menopause^12^, ^21^ Women are affected by various physiological cycles^19^ Great differences in body structure^10^ Women report lower levels of fertility education than men^11^, ^21^ Women Involvement in the whole process of pregnancy and childbirth causes more psychological trauma, perspectives on gender and role identity influencing cancer‐related infertility^10^	"Men vs Women: Dissecting the gender disparity in the perception and management of fertility problems"	
*"Beyond biology: The role of cultural stereotypes in gendered perceptions of fertility"* 10, 11 Gender stereotypes about females, pregnancy, and childrearing, with infertility described as a greater psychological blow to women^10^ Being pregnant was positioned as central to the desire for parenthood for some women^13^ Threat to masculine identity:Inferior and impotent^10^, ^20^, ^21^		
*"Diverse focus points: Unpacking gender differences in fertility* 7, 11, 13 Males discussed the importance their female partners' (non‐cancer survivors) placed on childbearing and parenthood^5^, ^6^, ^7^, ^13^ Clinical job satisfaction is associated with the presence or absence of fertility discussions^12^, ^15^ Men can learn about their fertility before treatment but often avoid fertility assessments afterwards^13^ Females reported feeling pressure related to dating and others' expectations^7^ Men focused primarily on when rather than‘if fertility would return^13^, ^19^ Men are concerned about loss of control and their partner's reaction to infertility women experience infertility as a direct blow to their self‐identity^13^		
*"Gender differences in managing fertility struggles"* 6, 10, 11, 13, 19 Women are more open to reproductive issues^6^, ^18^ Women need more information about childbirth and a higher level of understanding^10^, ^11^, ^12^, ^19^ Women tend to be persistently concerned^5^, ^7^, ^8^, ^13^, ^16^ Men are more embarrassed when explaining fertility issues^13^, ^20^ Men are concerned about loss of control and their partner's reaction to infertility women experience infertility as a direct blow to their self‐identity^13^ Decision making about fertility preservation: Men most accepted^21^ Women to share a more intimate bond with a child than do men, Men were more apt to share the emotional significance of fertility in their lives^10^		
Attitude: Negative emotional experiences (Restricted by age)^7^, ^8^, ^11^, ^15^, ^21^ Demands: Focus on the future of intimacy^7^, ^10^, ^19^ More guidance and support^15^, ^19^ Necessary involvement of parents^11^, ^19^ Coping methods: Interaction with professionals^10^, ^20^, ^21^ Parental assistance^11^, ^20^ Versatile processing:positive,^8^, ^15^, ^20^ passive (escape)^8^, ^11^, ^15^, ^20^	Peering into young minds: Perception and response to fertility issues among pediatric and adolescent cancer patients	"The influence of age: Experiences with fertility issues among cancer patients at different life stages"
Attitude: Diverse expressions of concern: Own fertility and parenting ability,^2^, ^3^, ^5^, ^9^, ^21^ breastfeeding,^3^ health of the fetus,^4^ own health,^2^, ^4^ children's health and childcare,^2^, ^3^ intimate relationship^2^, ^10^, disease prognosis and survival,^20^ fertility and breastfeeding, sex life^7^, ^10^ Various emotions: multiple burdens of emotional interweaving,^1^ guilt (parents, spouse and family),^3^ remorse and self‐blame,^4^ fear of death shadow over life,^5^ loss of womanhood^6^, ^12^, ^15^ Demands: Making fertility preservation decisions^2^, ^5^, ^6^, ^8^, ^14^ Find professional help^4^, ^15^, ^16^, ^17^ Finding a balance between treatment, fertility and quality of life^12^, ^13^, ^19^ Coping methods: For more information and support from interested parties^2^, ^3^, ^4^, ^5^, ^9^, ^12^, ^14^ Adjusting expectations^5^, ^7^, ^9^ Self‐state regulation^2^, ^5^, ^6^, ^8^, ^14^: Seek medical support face anxiety squarely adjust mentality to overcome anxiety,^2^, ^8^ change bad lifestyle,^2^ improve self‐health awareness,^2^, ^6^ accept reality,^7^, ^8^, ^19^ confronting the worries,^18^ sharing the possible risk of infertility with partners^7^, ^10^ Balance between thearpy, fertility and quality life^2^, ^3^, ^4^, ^5^, ^9^, ^12^, ^14^	Facing the challenge: Perception and strategies for handling fertility issues in cancer patients of other ages	
Age^11^, ^20^ Different emotional reactions^7^, ^8^, ^10^, ^11^, ^15^, ^20^ Greater focus on relationships^7^, ^10^, ^11^, ^19^, ^21^ Parental participation^15^, ^20^ Communication challenges^10^, ^15^	The age gap: Differences in understanding and addressing fertility issues across age groups of cancer patients	
The struggle with cancer diagnosis and fertility concerns: Uncertainties about future fertility lack of awareness^20^ Higher emotional response^12^, ^20^ Multiple negative emotions^3^, ^4^, ^12^, ^13^, ^18^ Decision making for fertility protection measures: Consider the details of fertility,^1^, ^3^, ^4^, ^12^, ^18^, ^21^ repaid decision,^12^, ^17^, ^18^ early communication and don‘t to make assumptions^16^, ^21^ Networking with professionals^4^, ^18^, ^20^, ^21^ Assessment of the safety, feasibility, success rates, potential risks, and side effects of these measures^16^, ^20^	Pre‐Treatment: Navigating fertility ambiguities in the shadow of cancer	"The impact of treatment stages on fertility concerns: The evolution of perception and coping strategies in the course of cancer treatment"
Physiological changes and adjustments to fertility desires: Physiological adjustments: Change in treatment plan,^18^ seeking support^12^, ^18^ Mental adjustments: (Worrying about the future,^18^ dilemmas and concerns,^18^ particularly vulnerable,^18^ conceiving of a new future^9^) Finding psychological support, such as counseling or participating in a support group^12^, ^18^ Acquisition and updating of fertility information: New demands(fertility preservation efficacy,^3^, ^12^, ^19^ opportunities for post‐treatment fertility, and related issues,^3^, ^13^ side effects from treatment cause patients to focus on the present rather than fertility issues^19^)	During treatment: Seeking fertility pathways amidst the whirlpool of life	
Challenges and worries about recovering fertility capabilities: Fertility restoration and self‐reproductive status^10^, ^11^ Seeking balance between hope and suffering: Experiencing pain while being full of hope^5^, ^7^, ^9^ Needs of female patients who are pregnant or planning pregnancy: Additional support and attention(plans for pregnancy,^16^ sharing the possible risk of infertility with partners,^11^ need for information on possible loss of fertility^10^, ^11^) Actively seeking support^7^, ^16^ Learning to cope with oneself^2^, ^5^, ^16^ Need for psychological support^16^	Post‐Treatment: Exploring the future of fertility amid hope and concern	

## References

[1] Sung H , Ferlay J , Siegel RL , et al. Global Cancer Statistics 2020: GLOBOCAN estimates of incidence and mortality worldwide for 36 cancers in 185 countries. CA Cancer J Clin. 2021;71(3):209‐249. 33538338 10.3322/caac.21660

[2] Cao MM , Chen WQ . Interpretation on the global cancer statistics of GLOBOCAN 2020. Chinese Journal of the Frontiers of Medical Science(Electronic Version). 2021;13(03):63‐69.

[3] Zheng R , Zhang S , Zeng H , et al. Cancer incidence and mortality in China, 2016. J Natl Cancer Center. 2022;2(1):1‐9. 10.1016/j.jncc.2022.02.002 PMC11256658 39035212

[4] Mosele F , Remon J , Mateo J , et al. Recommendations for the use of next‐generation sequencing (NGS) for patients with metastatic cancers: a report from the ESMO precision medicine working group. Ann Oncol. 2020;31(11):1491‐1505. 32853681 10.1016/j.annonc.2020.07.014

[5] Aravanis AM , Lee M , Klausner RD . Next‐generation sequencing of circulating tumor DNA for early cancer detection. Cell. 2017;168(4):571‐574. 28187279 10.1016/j.cell.2017.01.030

[6] Seashore‐Ludlow B , Rees MG , Cheah JH , et al. Harnessing connectivity in a large‐scale small‐molecule sensitivity dataset. Cancer Discov. 2015;5(11):1210‐1223. 26482930 10.1158/2159-8290.CD-15-0235 PMC4631646

[7] Zeng H , Chen W , Zheng R , et al. Changing cancer survival in China during 2003–15: a pooled analysis of 17 population‐based cancer registries. Lancet Glob Health. 2018;6(5):e555‐e567. 29653628 10.1016/S2214-109X(18)30127-X

[8] Miller KD , Fidler‐Benaoudia M , Keegan TH , Hipp HS , Jemal A , Siegel RL . Cancer statistics for adolescents and young adults, 2020. CA Cancer J Clin. 2020;70(6):443‐459. 32940362 10.3322/caac.21637

[9] Bray F , Ferlay J , Soerjomataram I , Siegel RL , Torre LA , Jemal A . Global cancer statistics 2018: Globocan estimates of incidence and mortality worldwide for 36 cancers in 185 countries. CA Cancer J Clin. 2018;68(6):394‐424. 30207593 10.3322/caac.21492

[10] Griffiths MJ , Winship AL , Hutt KJ . Do cancer therapies damage the uterus and compromise fertility? Hum Reprod Update. 2020;26(2):161‐173. 31863097 10.1093/humupd/dmz041

[11] Mahajan N . Fertility preservation in female cancer patients: an overview. J Hum Reprod Sci. 2015;8(1):3‐13. 25838742 10.4103/0974-1208.153119 PMC4381379

[12] Zhu F , Lu Q , Liu CL , et al. Concept analysis of reproductive concerns among cancer patients. Med Res Educ. 2021;38(5):51‐58.

[13] Geue K , Richter D , Schmidt R , et al. The desire for children and fertility issues among young German cancer survivors. J Adolesc Health. 2014;54(5):527‐535. 24315429 10.1016/j.jadohealth.2013.10.005

[14] Xie J , Sun Q , Duan Y , et al. Reproductive concerns among adolescent and young adult cancer survivors: a scoping review of current research situations. Cancer Med. 2022;11(18):3508‐3517. 35332694 10.1002/cam4.4708 PMC9487873

[15] Gorman JR , Su HI , Roberts SC , Dominick SA , Malcarne VL . Experiencing reproductive concerns as a female cancer survivor is associated with depression. Cancer. 2015;121(6):935‐942. 25377593 10.1002/cncr.29133 PMC4352116

[16] Qiao TT , Yan P , Qu SP , et al. Correlation between reproductive apprehension and depression in young female cancer patients:a Grade III‐A hospital in Urumqi. Mod Prev Med. 2019;46(17):3134‐3138.

[17] An XH , Zhao WT , Zhang Y , et al. Research progress on reproductive concern of young breast cancer patients. Chin J Mod Nurs. 2020;7:972‐976.

[18] Ljungman L , Eriksson LE , Flynn KE , et al. Sexual dysfunction and reproductive concerns in Young men diagnosed with testicular cancer: an observational study. J Sex Med. 2019;16(7):1049‐1059. 31255211 10.1016/j.jsxm.2019.05.005

[19] Ren HL , Zhao Y , Jiao NN . Relationship among self‐disclosure and reproductive concerns in patients with breast cancer of childbearing age in a Grade‐A tertiary Hospital in Tianjin. Med Soc. 2022;35(4):95‐99.

[20] Benedict CC , Thom BB , Friedman DD , et al. Young adult female cancer survivors' unmet information needs and reproductive concerns contribute to decisional conflict about post‐treatment fertility preservation. Cancer. 2016;122(13):2101‐2109. 27213483 10.1002/cncr.29917 PMC4911318

[21] Bártolo A , Neves M , Carvalho B , et al. Fertility under uncertainty: exploring differences in fertility‐related concerns and psychosocial aspects between breast cancer survivors and non‐cancer infertile women. Breast Cancer. 2020;27(6):1177‐1186. 32583350 10.1007/s12282-020-01124-w

[22] Ruddy KJ , Gelber SI , Tamimi RM , et al. Prospective study of fertility concerns and preservation strategies in young women with breast cancer. J Clin Oncol. 2014;32(11):1151‐1156. 24567428 10.1200/JCO.2013.52.8877 PMC4164759

[23] Jiang XY , Xu XY , Hu LF . Analysis of the current status and influencing factors of fertility worries in postoperative chemotherapy patients with breast cancer. Nurs Pract Res. 2020;17(19):89‐91.

[24] Letourneau JM , Ebbel EE , Katz PP , et al. Pretreatment fertility counseling and fertility preservation improve quality of life in reproductive age women with cancer. Cancer. 2012;118(6):1710‐1717. 21887678 10.1002/cncr.26459 PMC3235264

[25] Clasen NHZ , Van Der Perk MEM , Neggers SJCMM , Bos AME , van den Heuvel‐Eibrink MM . Experiences of female childhood cancer patients and survivors regarding information and counselling on gonadotoxicity risk and fertility preservation at diagnosis: a systematic review. Cancers. 2023;15(7):1946. 37046607 10.3390/cancers15071946 PMC10093478

[26] Zhang HM , Sun XC , Ma HM . Psychological experience of reproductive concerns in young cancer patients:a qualitative meta‐synthesis. J Nurs. 2022;29(2):51‐56.

[27] Xiao YQ , Li JH , Lei J , et al. Meta‐integration of the qualitative studies on fertility counseling experience in breast cancer patients of childbearing age. Chin Nurs Manage. 2022;22(12):1837‐1843.

[28] Xing NF , Yao D , Wang LS , Li S , Wang G . Experience of fertility decision‐making in women of childbearing age with breast cancer:a meta‐synthesis. Chin Nurs Manage. 2023;23(5):719‐724.

[29] The global burden of childhood and adolescent cancer in 2017: an analysis of the global burden of disease study 2017. Lancet Oncol. 2019;20(9):1211‐1225. 31371206 10.1016/S1470-2045(19)30339-0 PMC6722045

[30] Siegel RL , Miller KD , Jemal A . Cancer statistics, 2020. CA Cancer J Clin. 2020;70(1):7‐30. 31912902 10.3322/caac.21590

[31] Dittrich R , Kliesch S , Schüring A , et al. Fertility preservation for patients with malignant disease. Guideline of the DGGG, DGU and DGRM (S2k‐Level, AWMF Registry No. 015/082, November 2017) – recommendations and statements for girls and women. Geburtshilfe Frauenheilkd. 2018;78(6):567‐584. 29962516 10.1055/a-0611-5549 PMC6018069

[32] Vakalopoulos I , Dimou P , Anagnostou I , Zeginiadou T . Impact of cancer and cancer treatment on male fertility. Hormones (Athens). 2015;14(4):579‐589. 26732148 10.14310/horm.2002.1620

[33] Benedict C , Shuk E , Ford JS . Fertility issues in adolescent and young adult cancer survivors. J Adolesc Young Adult Oncol. 2016;5(1):48‐57. 26812452 10.1089/jayao.2015.0024 PMC4779291

[34] Newton K , Howard AF , Thorne S , Kelly MT , Goddard K . Facing the unknown: uncertain fertility in young adult survivors of childhood cancer. J Cancer Surviv. 2021;15(1):54‐65. 32613442 10.1007/s11764-020-00910-x

[35] Jardim FA , Lopes‐Júnior LC , Nascimento LC , Neves ET , de Lima RAG . Fertility‐related concerns and uncertainties in adolescent and young adult childhood cancer survivors. J Adolesc Young Adult Oncol. 2021;10(1):85‐91. 32945713 10.1089/jayao.2020.0058

[36] Ussher JM , Perz J , Australian Cancer and Fertility Study Team (ACFST) . Threat of biographical disruption: the gendered construction and experience of infertility following cancer for women and men. BMC Cancer. 2018;18(1):250. 29506492 10.1186/s12885-018-4172-5 PMC5836444

[37] Ruiz S , Mintz R , Sijecic A , et al. Websites about, not for, adolescents? A systematic analysis of online fertility preservation information for adolescent and young adult cancer patients. J Cancer Surviv. 2023. doi:10.1007/s11764-023-01386-1 37145331

[38] Logan S , Perz J , Ussher JM , Peate M , Anazodo A . Systematic review of fertility‐related psychological distress in cancer patients: informing on an improved model of care. Psychooncology. 2019;28(1):22‐30. 30460732 10.1002/pon.4927

[39] Lockwood C , Porritt K , Munn Z , et al. Chapter 2: systematic reviews of qualitative evidence. In: Aromataris E , Munn Z , eds. JBI manual for evidence synthesis. The Joanna Briggs Institute; Joanna Briggs Institute Reviewer's Manual; 2020.

[40] Tong A , Flemming K , Mcinnes E , Oliver S , Craig J . Enhancing transparency in reporting the synthesis of qualitative research: ENTREQ. BMC Med Res Methodol. 2012;12:181. 23185978 10.1186/1471-2288-12-181 PMC3552766

[41] Joanna Briggs Institute . Summary of findings tables for Joanna Briggs Institute Systematic Reviews. 2016 http://joannabriggs.org/assets/docs/sumari/Summary_of_Findings_Tables_for_Joanna_BriggsInstitute_Systematic_Reviews‐V3.pdf

[42] Xiao Y , Li J , Lei J , et al. Qualitative study of the fertility information support experiences of young breast cancer patients. Eur J Oncol Nurs. 2023;62:102275. 36716530 10.1016/j.ejon.2023.102275

[43] Wang B . A qualitative research on reproductive concerns of young cervical cancer patients with fertility‐sparing surgery. Zhejiang Chinese Medical University; 2022.

[44] Wang X , Wang Y , Pang J , et al. A qualitative study of fertility apprehension experiences in breast cancer patients of childbearing age. J Nurs Train. 2021;36(4):329‐332. +38.

[45] Tang L , Meng A , Hu T , et al. Qualitative research on the reproductive concerns of young female with thyroid cancer. Med Higher Vocat Educ Mod. 2020;3(3):195‐199.

[46] Azizi M , Ebrahimi E , Moghadam ZB , Shahhosseini Z , Modarres M . Pregnancy health among breast cancer survivors: development and validation of an educational package in Iran. J Cancer Educ. 2023;38:1373‐1382. 36856948 10.1007/s13187-023-02275-y

[47] Carr AL , Roberts S , Bonnell LN , Kolva E . Existential distress and meaning making among female breast cancer patients with cancer‐related fertility concerns. Palliat Support Care. 2022;21:1‐9. 10.1017/S1478951522001675 36562084

[48] Quinn GP , Murphy D , Knapp CA , Christie J , Phares V , Wells KJ . Coping styles of female adolescent cancer patients with potential fertility loss. J Adolesc Young Adult Oncol. 2013;2(2):66‐71. 23781403 10.1089/jayao.2012.0038 PMC3684136

[49] Vanstone RN , Fergus K , Ladhani NNN , Warner E . Reproductive concerns and fear of cancer recurrence: a qualitative study of women's experiences of the perinatal period after cancer. BMC Pregnancy Childbirth. 2021;21(1):738. 34717568 10.1186/s12884-021-04208-3 PMC8556905

[50] Wang Y , Logan S , Stern K , et al. Supportive oncofertility care, psychological health and reproductive concerns: a qualitative study. Support Care Cancer. 2020;28(2):809‐817. 31154532 10.1007/s00520-019-04883-1

[51] Perz J , Ussher J , Gilbert E . Loss, uncertainty, or acceptance: subjective experience of changes to fertility after breast cancer. Eur J Cancer Care. 2014;23(4):514‐522. 10.1111/ecc.12165 24372983

[52] Dryden A , Ussher JM , Perz J . Young women's construction of their post‐cancer fertility. Psychol Health. 2014;29(11):1341‐1360. 24916140 10.1080/08870446.2014.932790

[53] Kirkman M , Stern C , Neil S , et al. Fertility management after breast cancer diagnosis: a qualitative investigation of Women's experiences of and recommendations for professional care. Health Care Woman Int. 2013;34(1):50‐67. 10.1080/07399332.2012.735729 23216096

[54] Penrose R , Beatty L , Mattiske J , Koczwara B . Fertility and cancer—a qualitative study of Australian cancer survivors. Support Care Cancer. 2012;20(6):1259‐1265. 21660668 10.1007/s00520-011-1212-y

[55] Corney RH , Swinglehurst AJ . Young childless women with breast cancer in the UK: a qualitative study of their fertility‐related experiences, options, and the information given by health professionals. Psychooncology. 2014;23(1):20‐26. 24038590 10.1002/pon.3365

[56] Crawshaw MA , Sloper P . 'Swimming against the tide'—the influence of fertility matters on the transition to adulthood or survivorship following adolescent cancer. Eur J Cancer Care. 2010;19(5):610‐620. 10.1111/j.1365-2354.2009.01118.x 20088919

[57] Crawshaw MA , Glaser AW , Hale JP , et al. Male and female experiences of having fertility matters raised alongside a cancer diagnosis during the teenage and young adult years. Eur J Cancer Care. 2009;18(4):381‐390. 10.1111/j.1365-2354.2008.01003.x 19594609

[58] Chapple A , Salinas M , Ziebland S , McPherson A , Macfarlane A . Fertility issues: the perceptions and experiences of young men recently diagnosed and treated for cancer. J Adolesc Health. 2007;40(1):69‐75. 17185208 10.1016/j.jadohealth.2006.07.010

[59] Ma D . An overview of reproductive oncology. Chin J Pract Gynecol Obstet. 2018;34(12):1305‐1308.

[60] Wang R , Cheng R , Wang JX . Analysis on the status quo and influencing factors of reproductive anxiety in fertile patients with breast cancer. Chin Nurs Res. 2019;33(13):2258‐2261.

[61] Wang M , Zhu L , Xiong H , et al. Lack of knowledge, the main stumbling block of fertility preservation promotion in China. J Cancer Educ. 2022;37(3):739‐747. 32920747 10.1007/s13187-020-01875-2

[62] Drizin JH , Whitcomb BW , Hsieh T‐C , Gorman JR . Higher reproductive concerns associated with fertility consultation: a cross‐sectional study of young adult male cancer survivors. Support Care Cancer. 2020;29(2):741‐750. 32451700 10.1007/s00520-020-05527-5

[63] Young K , Shliakhtsitsava K , Natarajan L , et al. Fertility counseling before cancer treatment and subsequent reproductive concerns among female adolescent and young adult cancer survivors. Cancer. 2019;125(6):980‐989. 30489638 10.1002/cncr.31862 PMC6402962

[64] Liu Q , Xing L , Wang CY , et al. Current situation and prospect of fertility preservation in male cancer patients. J Chin Phys. 2017;4:481‐486.

[65] Chen YY , Wu KJ . Status and clinical response of fertility preservation in young patients with breast cancer. Chin J Surg. 2021;2:104‐108. 10.3760/cma.j.cn112139-20201013-00750 33378801

